# Effect of diet low in omega-6 polyunsaturated fatty acids on the global burden of cardiovascular diseases and future trends: evidence from the Global Burden of Disease 2021

**DOI:** 10.3389/fmed.2024.1485695

**Published:** 2025-01-07

**Authors:** Qingsong Mao, Yuzhe Kong

**Affiliations:** ^1^Hepatobiliary Pancreatic Surgery, Banan Hospital Affiliated of Chongqing Medical University, Banan, China; ^2^Xiangya School of Medicine, Central South University, Changsha, China

**Keywords:** cardiovascular disease, omega-6 polyunsaturated fatty acids, mortality forecasting, epidemiology, disease burden

## Abstract

**Background:**

This research analyzes the worldwide impact of cardiovascular diseases (CVD) associated with low consumption of omega-6 polyunsaturated fatty acids, utilizing data from the 2021 Global Burden of Disease Study.

**Method:**

The study explored the influence of diets deficient in omega-6 polyunsaturated fatty acids on CVD across global, regional, and national levels. It examined variations across different age groups and genders and analyzed the relationship between the disease burden and the socio-demographic index (SDI). Furthermore, it employed an ARIMA model to project the future prevalence of CVD linked to insufficient omega-6 intake until 2050.

**Result:**

In 2021, insufficient omega-6 intake was linked to roughly 737.88 thousand deaths and 17.87 million disability-adjusted life years (DALYs) due to CVD, showing a decreasing trend in this health burden throughout the study period. The most significant effects were seen in individuals aged 75 and older, with a higher disease burden noted in males. Forecasts suggest likely declines in disease prevalence in regions with high SDI. On a national level, regions like Russia and various countries in North Africa and the Middle East might experience increasing challenges related to CVD due to low omega-6 intake by 2030 and 2050.

**Conclusion:**

These results highlight the critical need for preventive strategies for CVD and stress the importance of managing dietary patterns to mitigate health risks.

## Graphical abstract

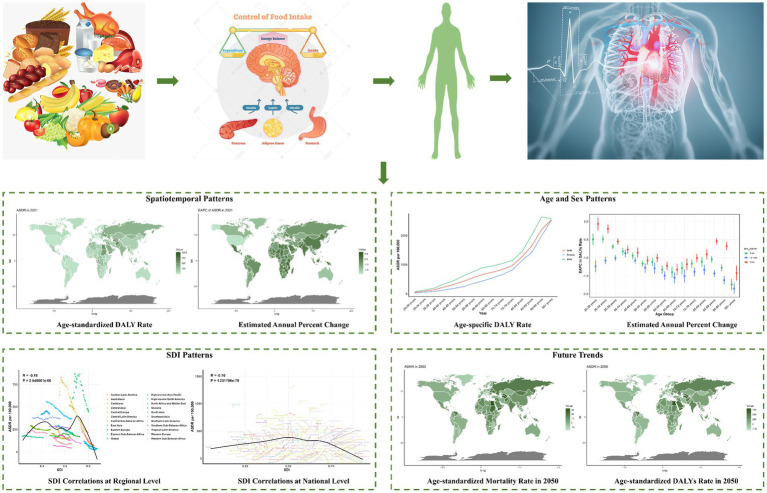

## 1 Introduction

Cardiovascular diseases (CVD) are the primary cause of mortality worldwide (1). Numerous risk factors including smoking, dyslipidemia, hypertension, obesity, lack of physical activity, and ethnicity are closely associated with CVD (1). It is noteworthy that behavioral risk factors could prevent up to 90% of CVD incidents (2). Additionally, dietary habits and specific dietary components significantly influence CVD (3). LA, a crucial fatty acid that must be ingested through diet sources like vegetable and nut oils, poultry, meat, eggs, milk, and margarine, cannot be synthesized by humans (4, 5). AA, derived from LA, is also a key PUFA (6, 7).

Growing research highlights the potential impact of omega-6 PUFAs on reducing CVD risk (8–10). For instance, a meta-analysis found that higher LA levels in the body were linked to a reduced risk of coronary heart disease (CHD) (11). However, no significant association was found between AA levels and CHD risk (8). Another analysis demonstrated that increasing dietary LA by 5% of total energy, substituting saturated fats, correlated with a 9% reduction in CHD incidents (9). A comprehensive analysis from 11 studies indicated that omega-6 PUFAs were associated with a decrease in both CHD events and mortality, showing hazard ratios of 0.87 and 0.74, respectively (8). Another study showed replacing saturated fats with omega-6 PUFAs significantly cut total cholesterol by 19%, LDL-C by 22%, and increased HDL-C by 14% among nutrition students (9). Further, each 5% replacement of saturated fats with PUFAs reduced LDL-C by 10 mg/dL and the TC ratio by 0.16 (12).

Despite these findings, some studies offer mixed results. The Sydney Diet Heart Study, an RCT with 458 middle-aged men with recent coronary events, reported increased mortality rates when replacing saturated fats with LA (13). Additionally, comprehensive reviews have found no definitive links between omega-6 PUFA intake and CVD outcomes (14, 15). It is suggested that diets high in omega-6 PUFAs might elevate proinflammatory mediators like prostaglandins and leukotrienes, which could adversely affect CVD risk (4, 16).

The limitations of individual studies, including small sample sizes, variations in research design, and diverse participant profiles, underline the need for broader investigations. This study utilized the 2021 Global Burden of Disease data to explore the effects of a diet low in omega-6 PUFAs on CVD. Moreover, future CVD trends were predicted using the ARIMA model, validated by previous research (17, 18).

## 2 Method

### 2.1 Study population

Our investigation drew upon the Global Burden of Disease Study 2021, sourced from http://ghdx.healthdata.org/gbd-results-tool, encompassing assessments of 369 health conditions and the influence of 87 factors across 204 nations over the period from 1990 to 2021 (19).

The study elaborates on methodologies applied to gauge the effects of cardiovascular diseases (CVD). The detailed methods are outlined in referenced studies (20, 21). CVD mortality data were collected from various sources like vital registration systems and health surveys, among others, and were adjusted to account for inaccuracies and misclassifications (22). These adjusted data were fed into the Cause of Death Ensemble model (CODEm) to produce annual CVD mortality estimates by age and sex for each region (21, 23). Additionally, a comparative risk assessment pinpointed key factors contributing to CVD, with the population attributable fraction (PAF) being calculated to measure the impact of a diet low in omega-6 polyunsaturated fatty acids on CVD (19). Both mortality and disability-adjusted life years (DALYs) associated with this dietary risk were assessed, where DALYs are comprised of years lost due to premature death (YLLs) and years lived with disability (YLDs). YLD calculations considered the prevalence of these infections, adjusted by disability severity (22). The socio-demographic index (SDI) was computed considering factors such as fertility rates in individuals under 25 (TFU25), educational attainment for those aged over 15 (EDU15+), and adjusted *per capita* income. Countries were categorized into five SDI levels: low, low-middle, middle, high-middle, and high (22, 24). In our studies, we used mortality and DALYs to estimate the risks.

### 2.2 Definition of diet low in omega-6 polyunsaturated fatty acids

A diet low in omega-6 polyunsaturated fatty acids is characterized by consuming less than 9–10% of total daily energy from sources such as linoleic acid, γ-linolenic acid, eicosadienoic acid, dihomo-γ-linolenic acid, and arachidonic acid.^1^

### 2.3 Statistical analysis

Our statistical analysis involved standardizing mortality and DALY rates for international comparisons using age-adjusted rates (AAR) to normalize for age distribution and demographic differences among countries. We employed a linear regression on the logarithmic values of these rates, expressed as *y* = *α* + *βx* + *ε*, where *x* denotes the year. The estimated annual percentage change (EAPC) was derived using the equation 100 * (e^β^ − 1), along with its 95% confidence interval (95% CI). Trends in AAR were categorized as increasing, decreasing, or stable based on the direction and magnitude of the EAPC and the limits of the 95% CI (25, 26). The relationship between AAR and SDI was explored using Gaussian process regression with Loess smoothing and analyzed through Spearman rank correlation tests (24, 26, 27).

Additionally, the ARIMA model was applied to examine and forecast the influence of low omega-6 polyunsaturated fatty acid diets on CVD trends from 2020 to 2050. This model’s configuration (p, d, q) includes “AR” for the autoregressive component, “MA” for the moving average terms, and “d” for the degree of differencing needed for data stability (28). The model’s efficacy was judged based on the Akaike information criterion (AIC) and the Bayesian information criterion (BIC).

All metrics were calculated with 95% uncertainty intervals and presented per 100,000 individuals. Data handling, analysis, and visualization were conducted using R software, version 4.3.2 (29–32).

## 3 Result

### 3.1 Spatiotemporal patterns of CVD attributable to diet low in polyunsaturated fatty acids

In 2021, a diet deficient in omega-6 polyunsaturated fatty acids contributed to approximately 737,880 deaths [percentage (95% UI): 3.78 (−11.29, 14.64)] and 17.87 million disability-adjusted life years (DALYs) [percentage (95% UI): 4.15 (−13.48, 15.59)] from cardiovascular diseases (CVDs). The age-standardized mortality rate (ASMR) was 8.7966 per 100,000 population, with a 95% uncertainty interval (UI) ranging from −25.1372 to 33.0790, and the age-standardized DALY rate (ASDR) was 208.1459 per 100,000, with a 95% UI from −651.9420 to 768.8355. Over the last 30 years, there has been a significant reduction in the impact of diets low in omega-6 polyunsaturated fatty acids on CVDs (Table 1; Supplementary Table S1).

**Table 1:** Global and regional deaths and DALYs of CVD attributable to diet low in omega-6 polyunsaturated fatty acids in 1990 and 2021 in 27 global regions.

Location	Deaths number in 1990	Deaths number in 2021	ASMR in 2021	DALY number in 1990	DALY number in 2021	ASDR in 2021
Global	444092.5961 (1671175.0823, −1329120.1503)	737879.2822 (2767688.4274, −2118790.2988)	8.7966 (33.0790, −25.1372)	11394660.9702 (42146675.3499, −37434183.6174)	17869860.5395 (65962498.5240, −56003352.1985)	208.1459 (768.8355, −651.9420)
Region						
East Asia	57078.3111 (217395.0256, −174264.1001)	166872.9602 (659447.6591, −454038.5936)	8.8171 (34.9010, −23.8510)	1588871.6800 (5963670.0046, −5216379.5287)	3453573.3006 (13297835.1233, −10054895.9990)	169.5589 (654.8998, −496.6453)
Southeast Asia	26630.8147 (99272.7060, −87664.2431)	64281.4401 (238038.8218, −195592.4708)	10.4271 (38.3311, −30.6034)	804757.6255 (2969452.9696, −2876452.0730)	1805964.2513 (6651800.8183, −5965544.1693)	259.3193 (959.2584, −831.5581)
Oceania	441.2740 (1630.3329, −1427.9436)	1178.6278 (4378.6318, −3908.8978)	15.7598 (58.5724, −48.8838)	14573.0553 (52711.7222, −49662.7171)	38570.6765 (141624.8710, −136383.4000)	421.1015 (1562.3570, −1403.8548)
Central Asia	12308.2388 (45947.3759, −37193.2137)	15881.8579 (59601.9689, −45979.8712)	22.5759 (85.2580, −62.9325)	298401.0824 (1100660.4376, −985763.0899)	376233.3502 (1416816.6129, −1166921.4690)	464.7148 (1743.6853, −1392.6879)
Central Europe	32086.8730 (121631.6425, −93034.1972)	24834.8262 (93323.0282, −67065.8039)	10.7757 (40.2516, −29.4781)	740620.5178 (2748370.7697, −2318934.9821)	458022.2390 (1693211.8623, −1294469.7822)	214.5585 (790.0062, −622.0477)
Eastern Europe	70208.8216 (266650.3256, −203037.3657)	74973.3908 (289423.0925, −207280.8060)	21.2563 (81.9802, −59.4298)	1581047.3533 (5890477.8389, −4964465.2598)	1513606.7048 (5761080.8364, −4452072.9379)	445.3967 (1683.5062, −1337.8134)
High-income Asia Pacific	8905.5271 (34232.8610, −24146.1780)	9188.0006 (36009.3985, −22699.1521)	1.6560 (6.3940, −4.2722)	191241.3995 (713486.2556, −555075.2388)	153526.9102 (588542.1855, −398786.6506)	36.4080 (136.6251, −100.8406)
Australasia	3399.9944 (13133.6035, −9517.6150)	2239.7768 (8574.6686, −6141.3567)	3.8029 (14.4098, −10.6609)	70426.7125 (267411.4159, −207427.8043)	39274.0621 (147292.5967, −113824.7034)	76.2392 (283.7637, −227.9293)
Western Europe	62845.7441 (243423.9324, −166591.5249)	34063.1735 (132509.4774, −85463.5945)	3.1387 (12.0975, −8.0430)	1249448.2961 (4740210.3859, −3459617.5953)	574733.9939 (2190138.5699, −1495443.4654)	63.8954 (239.4347, −172.8155)
Southern Latin America	5078.8335 (19471.3257, −14178.2591)	3528.7310 (13363.1520, −9271.7250)	3.9771 (15.0029, −10.5224)	115061.9141 (431685.7868, −341908.5560)	75738.7217 (282413.7753, −210622.5659)	89.0324 (330.5836, −250.6815)
High-income North America	19634.2812 (75655.6073, −45294.0754)	8389.6153 (33418.7174, −18530.4308)	1.2003 (4.7425, −2.6958)	398377.7153 (1534542.0941, −933775.2592)	151244.4998 (589982.2543, −351441.9006)	24.4182 (94.6531, −57.7335)
Caribbean	3193.1667 (12236.2499, −8772.5733)	3233.4327 (11894.2258, −8370.1303)	5.9303 (21.7806, −15.3900)	76418.9382 (284525.8580, −222796.8840)	75847.8721 (282874.6363, −209708.4161)	141.1186 (525.8974, −391.8139)
Andean Latin America	1347.4503 (5187.9282, −3778.8523)	2340.1767 (9250.9669, −6513.9819)	4.0541 (16.1023, −11.2238)	34714.8482 (130307.5646, −104982.9575)	55667.9087 (214107.4752, −160992.5141)	91.8361 (354.9692, −263.4889)
Central Latin America	6981.2503 (26447.1268, −19736.2040)	18065.8098 (66846.6023, −48008.5184)	7.4382 (27.6332, −19.6650)	179722.2069 (658197.8627, −552111.8571)	420101.2868 (1534064.1004, −1183325.1438)	165.4543 (604.8391, −461.7793)
Tropical Latin America	2639.0866 (10299.3961, −5850.8257)	4305.7952 (16595.9617, −9753.5791)	1.6826 (6.5016, −3.8032)	74589.6703 (282502.4871, −172403.2506)	117176.1807 (440904.4467, −270229.4111)	44.7335 (168.9970, −103.0821)
North Africa and Middle East	35895.1804 (134675.6040, −107804.8515)	66121.4715 (246493.2249, −199672.7731)	15.9564 (59.8216, −46.8758)	1018654.8707 (3761935.9742, −3295032.3242)	1788213.4971 (6665733.7862, −5657906.7177)	364.2772 (1349.4157, −1119.5503)
South Asia	79190.8618 (297847.9876, −276490.5766)	203918.5282 (762634.5493, −630311.7965)	14.4146 (54.0086, −43.0383)	2507057.2291 (9230836.6159, −9243275.9355)	5815228.8878 (21316522.3838, −19506065.3708)	367.9228 (1361.1013, −1198.5164)
Central Sub-Saharan Africa	2400.1836 (9710.5635, −7967.8824)	5128.5511 (19944.5000, −15480.6590)	10.8571 (42.7530, −32.0743)	68224.1462 (276929.5345, −239235.9499)	147249.1425 (567194.8437, −459968.8758)	247.9823 (961.9197, −748.8270)
Eastern Sub-Saharan Africa	4663.2752 (17738.6059, −15220.8996)	10379.8688 (38426.7051, −33250.9556)	6.7156 (25.2162, −20.1383)	139312.1912 (523913.0162, −484309.1792)	306794.1644 (1114876.0657, −1032015.0079)	160.8220 (595.4362, −517.9902)
Southern Sub-Saharan Africa	1634.7181 (5985.9750, −5015.2814)	2898.5886 (10778.0158, −8082.1706)	5.6563 (21.2105, −15.3363)	47861.7344 (174120.7710, −157682.1480)	79054.1816 (289848.7760, −234319.0241)	130.1331 (479.1072, −373.4885)
Western Sub-Saharan Africa	7528.7095 (28483.3616, −22950.8546)	16054.6597 (59683.2864, −52923.9657)	9.6362 (36.0891, −29.5376)	195277.7830 (721421.5905, −630572.4276)	424038.7078 (1573620.0795, −1511283.4838)	206.8922 (770.8895, −690.8502)
SDI						
High-middle SDI	142004.3025 (534797.3206, −418035.5134)	197178.3516 (749585.9151, −536582.1789)	10.2418 (38.9257, −27.9579)	3369120.7630 (12488662.1660, −10753841.5912)	4041034.1821 (15188900.2771, −11955260.0204)	210.2076 (788.6103, −629.1881)
High SDI	102346.3939 (393212.7641, −272352.3029)	66658.9027 (257559.0422, −171134.1479)	2.9240 (11.1074, −7.7349)	2133064.4534 (8043489.4444, −5964312.0000)	1244887.5572 (4676020.0382, −3415706.7033)	64.4118 (240.1901, −184.6085)
Low-middle SDI	77627.9201 (292627.4661, −262511.8328)	178538.6126 (658242.1943, −547477.1606)	12.9802 (48.4548, −38.3386)	2365797.0116 (8746888.3745, −8497857.4272)	5047367.6364 (18544434.5333, −16776813.2502)	327.2741 (1204.8922, −1054.3673)
Low SDI	24593.5197 (93665.0305, −80403.4978)	50640.0314 (184833.7428, −157498.9862)	10.9037 (40.0173, −32.4430)	728671.7984 (2736480.7893, −2516856.4355)	1459985.0854 (5307565.7496, −4824947.6445)	260.9715 (951.7984, −825.2166)
Middle SDI	96756.9709 (361279.2554, −306765.5333)	244148.6921 (910453.4654, −705346.8917)	9.8848 (36.9915, −27.7828)	2779776.8024 (10277395.3273, −9562134.5260)	6060649.4821 (22338521.6570, −19236036.8427)	224.8045 (830.4713, −701.6995)

**Figure 1:**
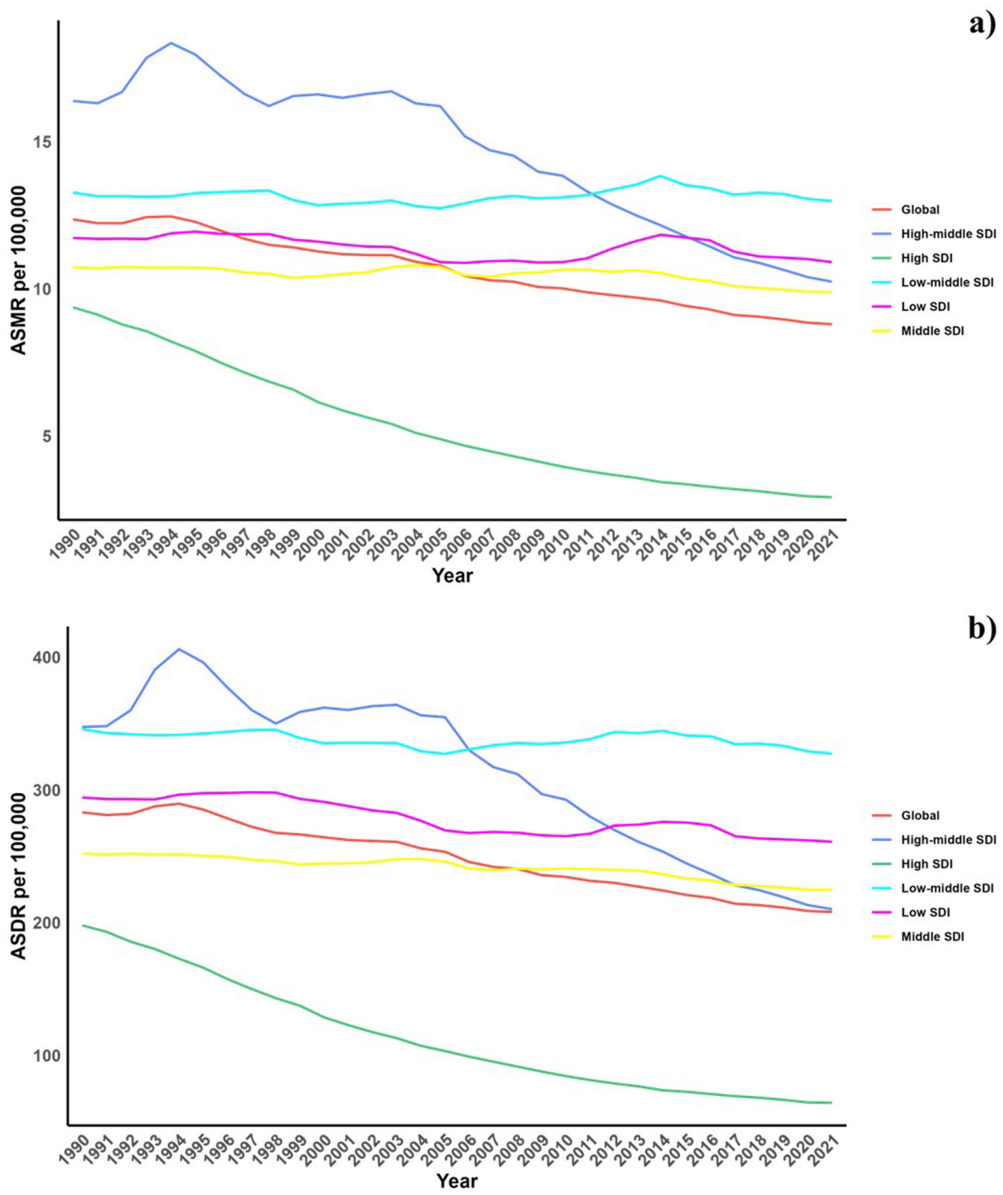
Temporal trends of ASMR and ASDR of CVD attributable to diet low in omega-6 polyunsaturated fatty acids from 1990 to 2021 in different SDI regions.

Regarding the socio-demographic index (SDI), higher SDI regions have seen a notable decline in CVD burden due to diets low in omega-6 fatty acids, with the largest decreases observed. Across all SDI categories, modest reductions were observed in both ASMR and ASDR for CVDs linked to these diets, with the exception of the high SDI regions (Table 1; Supplementary Table S1 and Figure 1).

On a regional level, the greatest burdens from diets low in omega-6 polyunsaturated fatty acids were found in Asia and Europe. In contrast, Oceania and Australia reported the lowest burdens associated with such diets (Table 1).

**Figure 2:**
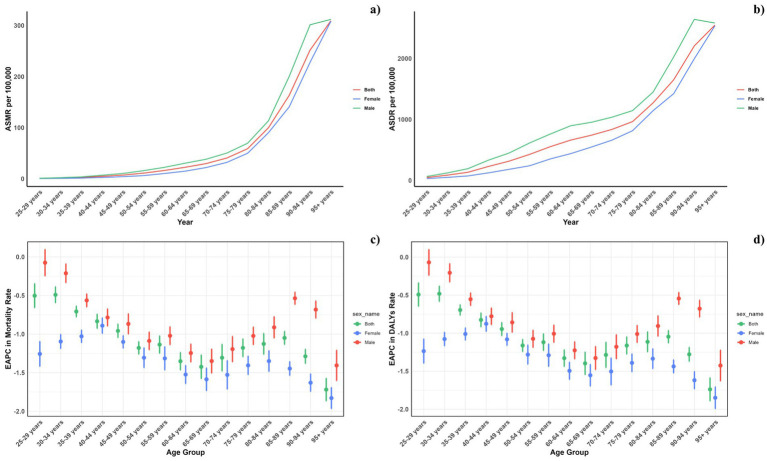
Age-specific rates of global deaths (a) and DALYs (b) of CVD attributable to diet low in omega-6 polyunsaturated fatty acids, by sex, in 2021 and the corresponding EAPC of global deaths (c) and DALYs (d) from 1990 to 2021.

**Figure 3:**
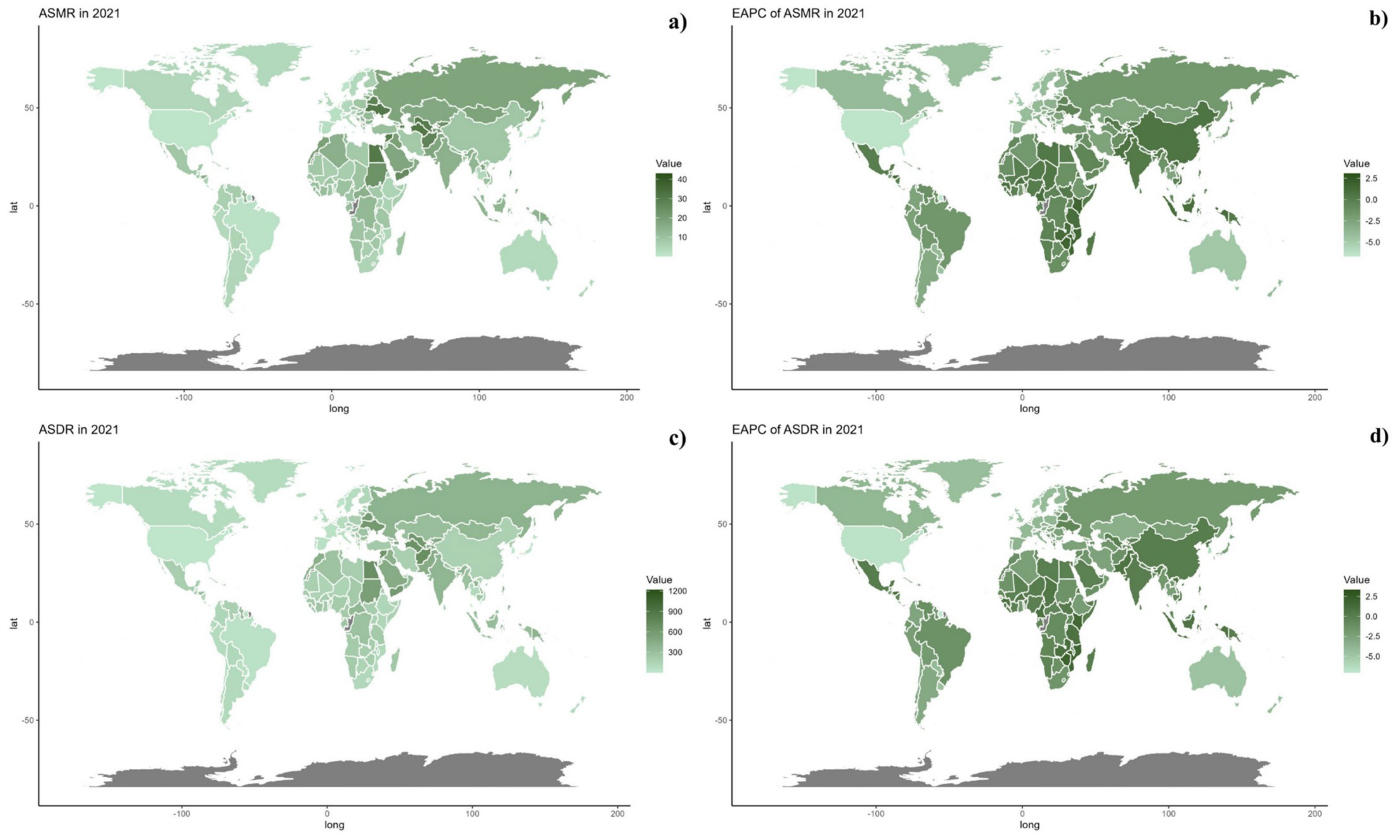
Global distribution of ASMR (a) and ASDR (c) of CVD attributable to diet low in omega-6 polyunsaturated fatty acids for both sexes in 2021 in 204 countries and territories. EAPC of ASMR (b) and ASDR (d) of CVD attributable to diet low in omega-6 polyunsaturated fatty acids from 1990 to 2021 in 204 countries and territories.

Nationally, in 2021, there was considerable global variation in the ASDR and ASMR for CVDs associated with omega-6 fatty acid-deficient diets. The highest rates were seen in North Africa, the Middle East, and Russia (Figure 2). From 1990 to 2019, a general trend of reductions in both ASMR and ASDR was noted across most countries, with the exception of those in North Africa and the Middle East (Table 1; Supplementary Table S1 and Figure 3).

### 3.2 Age and gender pattern

Figure 2 depicts the age-specific global mortality and DALY rates for cardiovascular diseases (CVDs) in 2021, along with their trends from 1990 to 2021. The graph demonstrates a J-shaped curve, indicating rising mortality and DALY rates in individuals under 75 years, with a marked increase observed in the 75 to 95 and older age group. Across all age groups, males consistently registered higher mortality rates due to diets low in omega-6 polyunsaturated fatty acids compared to females. Likewise, DALY rates linked to such diets were elevated across all ages for males. Moreover, the reduction in mortality and DALY rates from 1990 to 2021 was less substantial among males in every age group.

**Figure 4:**
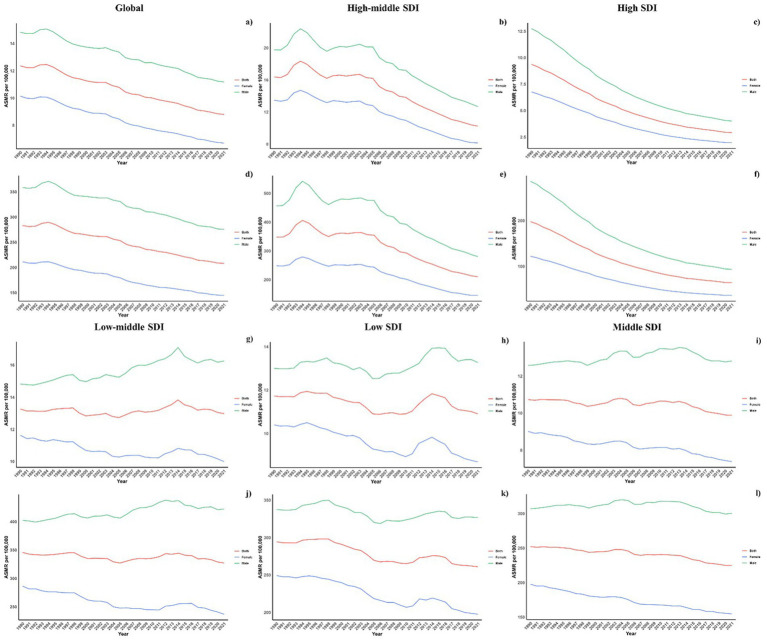
Sex disparity in CVD attributable to diet low in omega-6 polyunsaturated fatty acids across SDI regions.

Across all socio-demographic index (SDI) categories, male mortality and DALY rates were consistently higher, maintaining this gender disparity across the regions. However, in regions with high-middle and high SDI, the differences between genders have lessened, whereas in other SDI regions, the disparities have either remained unchanged or increased (Figure 4).

### 3.3 Association with the socio-demographic index

**Figure 5:**
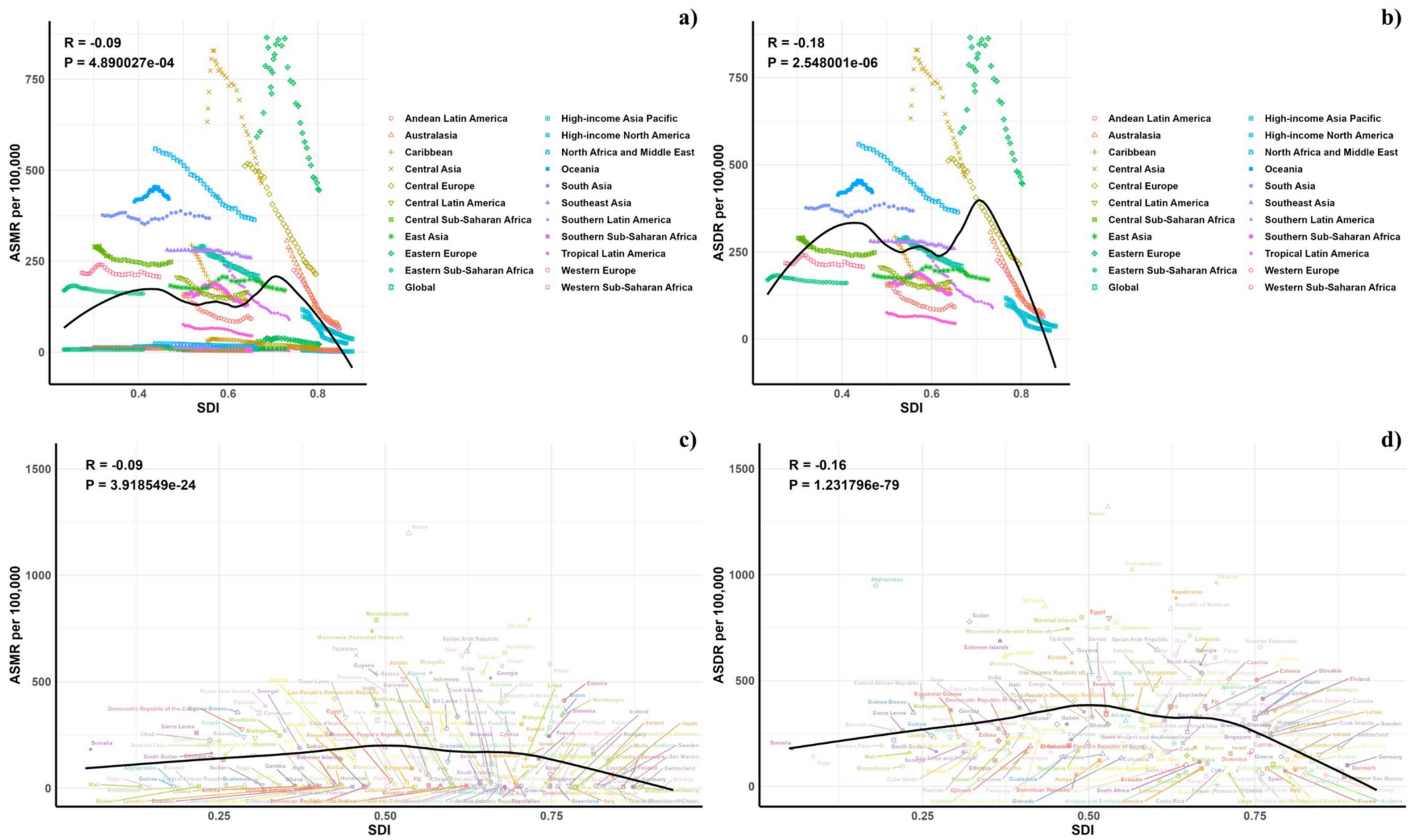
Correlations between ASMR (a,c) and ASDR (b,d) of CVD attributable to diet low in omega-6 polyunsaturated fatty acids and SDI at the regional level (a,b) and the national level (c,d).

Figure 5 displays a comparison between the actual and predicted age-standardized DALY (ASDR) and mortality rates (ASMR) for CVD related to diets low in omega-6 polyunsaturated fatty acids, aligning these with socio-demographic index (SDI) values at both regional and national levels from 1990 to 2021. A discernible negative correlation exists between ASDR and rising SDI, suggesting a decrease in disease burden as SDI increases. Regions such as Eastern Europe, Central Asia, North Africa, and the Middle East exhibited higher-than-expected ASDR during this period. The pattern of observed versus forecasted ASMR based on SDI at regional levels mirrors the ASDR findings. Figure 5 also demonstrates the observed versus anticipated ASDR and ASMR at the national level for 2021, showing a similar negative correlation with SDI values, both regionally and nationally.

### 3.4 Forecasts for the mortality, DALYs rate, ASMR and ASDR of CVD attributable to diet low in polyunsaturated fatty acids in global (2022–2050)

**Figure 6:**
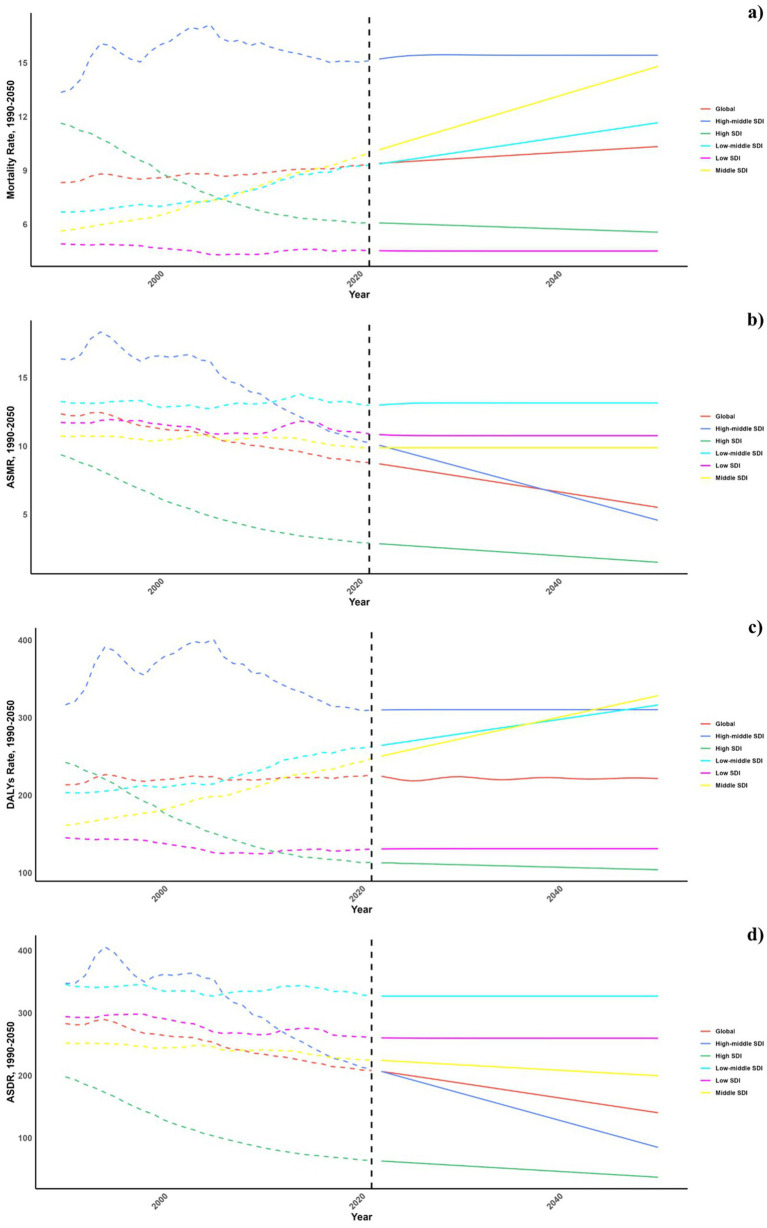
Estimated trends of mortality rate (a), DALYs Rate (b), ASMR (c) and ASDR (d), 1990–2050 at the regional level.

**Figure 7:**
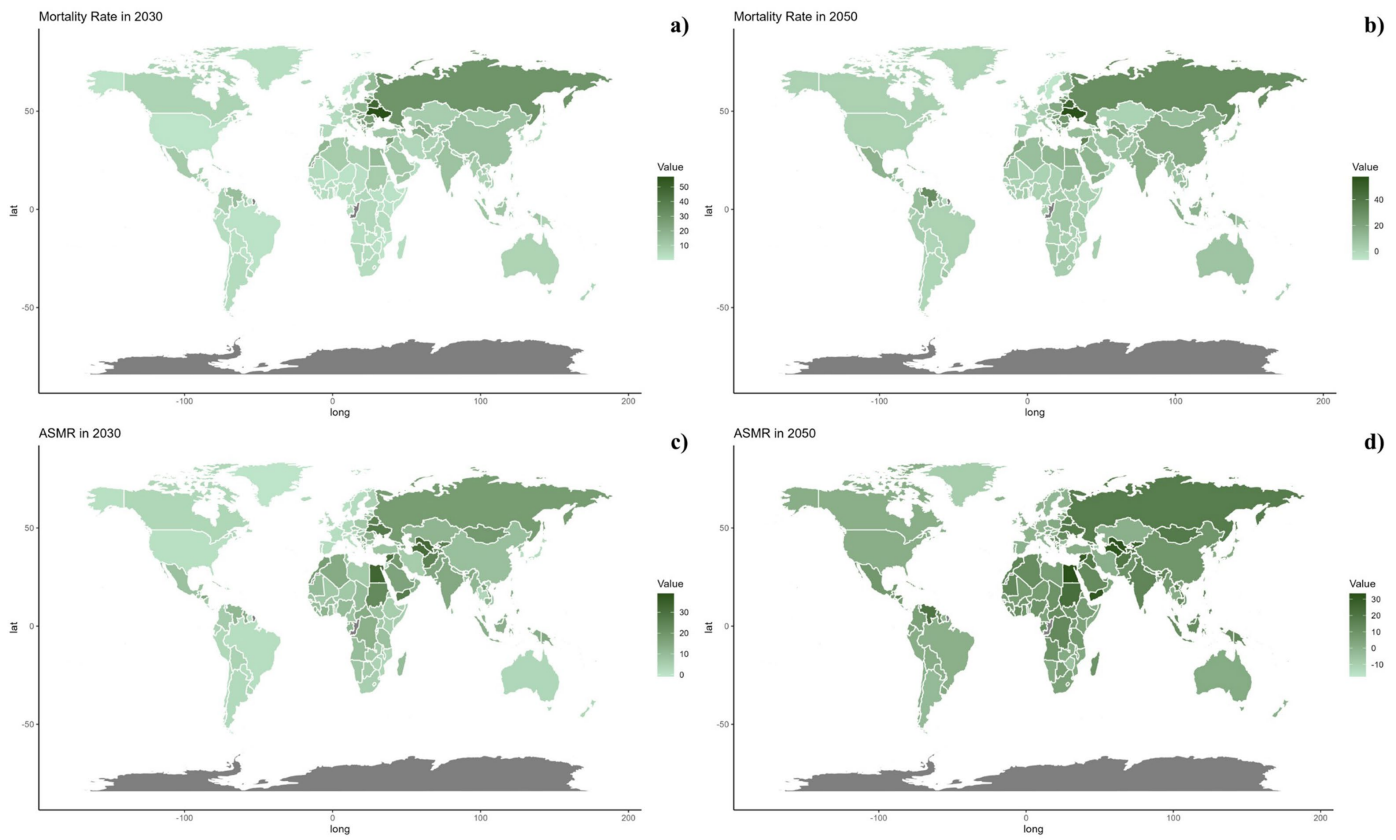
Estimated trends of mortality rate (a,b) and ASMR (c,d) in 2030 (a,c) and 2050 (b,d) at the national level.

**Figure 8:**
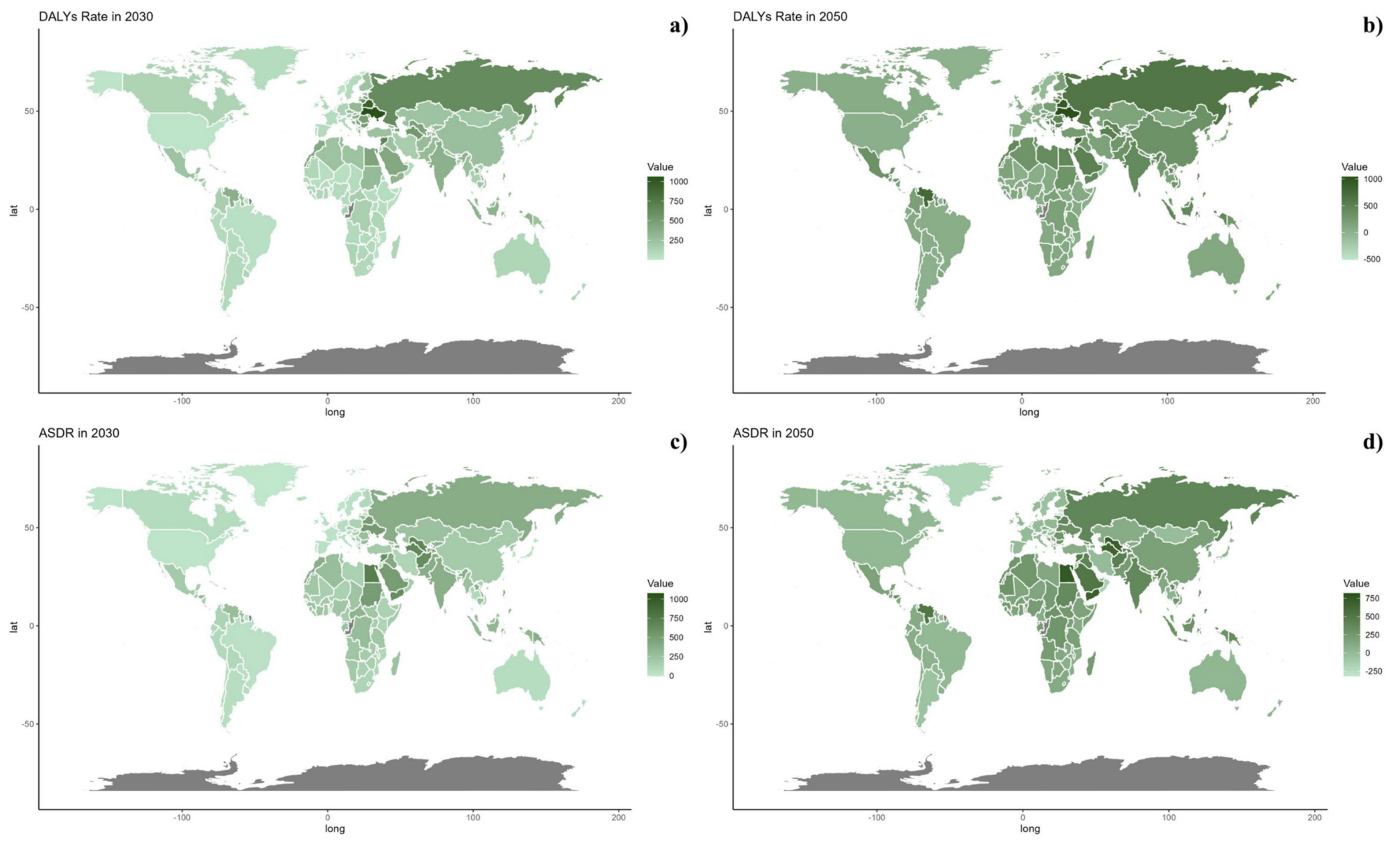
Estimated trends of DALYs rate (a,b) and ASDR (c,d) in 2030 (a,c) and 2050 (b,d) at the national level.

Figures 6, 7 outline the projected trends in mortality and DALY rates, including ASMR and ASDR for CVDs linked to diets deficient in omega-6 polyunsaturated fatty acids. At the regional scale, decreases in CVD burden are forecasted solely within the high and high-middle SDI regions, whereas other SDI regions are likely to maintain current rates regarding ASDR and ASMR. Middle and low-middle SDI regions, on the other hand, are projected to experience increases in both mortality and DALY rates (see Figure 8).

On a national scale, these patterns are expected to remain fairly consistent through the years 2030 and 2050. Nonetheless, the projected consequences of CVDs related to diets low in omega-6 polyunsaturated fatty acids are anticipated to be significantly greater in Russia and throughout the North Africa and Middle East region than in other regions for both 2030 and 2050.

## 4 Discussion

This investigation assessed the global consequences of cardiovascular disease (CVD) linked to diets deficient in omega-6 polyunsaturated fatty acids over the period from 1990 to 2021, identifying a significant decline. The most impacted demographic was males over the age of 75, who displayed elevated incidence rates. Projections for the future indicate a likely reduction in mortality rates in regions with higher social development index (SDI) scores, although high mortality and age-standardized death rates from such diets are expected to continue in Russia and the North Africa and Middle East region. This study represents the first extensive review of CVD effects stemming from insufficient intake of omega-6 polyunsaturated fatty acids.

Prior analyses, including a meta-analysis of 25 case-control studies, found a negative correlation between levels of linoleic acid (LA) in blood and tissues and coronary heart disease (CHD) risk (8). While arachidonic acid (AA) was not significantly linked to CHD risk, lower levels of AA in adipose tissue were tied to increased CHD event risk in longitudinal studies, yet were associated with a reduced risk in cross-sectional analyses (8). A different meta-analysis covering 13 cohort studies involving 30,602 subjects (with 12,479 CHD events, including 5,882 fatalities) highlighted the protective effects of increased LA intake against CHD occurrences and mortality, indicating that substituting saturated fats with LA could lower CHD event and death risks by 9 and 13%, respectively (9). Earlier analyses, including 60 clinical trials (33), and another encompassing 8 randomized control trials (RCTs) with 13,614 participants experiencing 1,042 CHD events (12), noted that omega-6 PUFAs positively influenced blood lipids and reduced CHD events. Moreover, a Cochrane review of dietary fat modifications through RCTs indicated that substituting saturated fats with monounsaturated or polyunsaturated fatty acids modestly but effectively decreased CVD risk over a minimum of 6 months (34).

A study of 1,551 middle-aged men explored the impact of dietary LA and total PUFA consumption on CVD mortality, revealing an inverse relationship with CVD deaths (35). A systematic review of 19 studies including 6,461 adults (average age: 50 years) suggested minimal effects on mortality or CVD events over 1–8 years by increasing omega-6 PUFA intake, though it could potentially lower heart attack risks (36). Omega-6 PUFAs were found to reduce serum total cholesterol (TC) by 6%, without significant changes to triglycerides (TG), high-density lipoprotein cholesterol (HDL-C), and low-density lipoprotein cholesterol (LDL-C). It is important to note that limited event numbers and the underrepresentation of participants from developing countries and women may have affected these findings (13).

Additional cohort research indicates that a diet low in saturated fatty acids and enriched with omega-6 PUFAs correlates with decreased CHD risk and lower levels of LDL-C (10, 11). Jakobsen et al. (11) demonstrated that substituting carbohydrates with PUFAs (omega-6) can reduce levels of TC, HDL-C, LDL-C, and TG. However, the validity of these findings is limited due to the short duration of interventions and the inclusion of non-randomized studies, which may undermine their clinical relevance (34).

The impact of omega-6 PUFAs on CVD risk remains debated. While some studies support the positive effects of omega-6 PUFA supplementation on cardiovascular outcomes, contrasting studies present divergent results. A potential explanation for the complex relationship between omega-6 PUFAs and CVD risk involves the enhanced synthesis of 2-series prostaglandins and 4-series leukotrienes, which are proinflammatory and may adversely influence residual CVD risk (4, 16). Furthermore, compounds like AA, a highly unsaturated fatty acid, could increase LDL-C and VLDL oxidation, elevating their atherogenic potential (4). Conversely, certain investigations show no correlation between omega-6 PUFA consumption and CVD risk, including a 14-year longitudinal study involving 43,732 men which found no link between overall fat consumption, cholesterol, and stroke risk (37). Additionally, an analysis of prospective observational studies and RCTs indicated that high PUFA intake coupled with low saturated fat intake did not significantly alter CVD outcomes (15). Likewise, a Cochrane review involving 660 participants detected no significant connections between varying omega-6 PUFA intake levels and CVD risk factors (14). Furthermore, another systematic review concluded that replacing saturated fats with cis-PUFAs, akin to omega-6 PUFAs, markedly reduced TC, supporting findings of our current study (38–46).

Previously, another study analyzes the global impact of nonoptimal dietary fat intake on coronary heart disease (CHD) mortality, highlighting the health burden linked to low ω-6 polyunsaturated fat (PUFA) intake and high saturated (SFA) and trans fats (TFA) consumption. It reveals significant disparities in CHD mortality, particularly in countries with poor dietary management and lower socioeconomic status. Targeted public health interventions, such as promoting ω-6-rich foods and reducing SFA and TFA intake, could substantially lower CHD rates, especially in high-risk regions. Additionally, regional variations indicate that global dietary guidelines should be tailored to local nutritional contexts. The study underscores the effectiveness of past interventions while revealing ongoing challenges in food policy. Ultimately, it provides a vital evidence base for future public health strategies to combat diet-related CHD worldwide. Future research should continue to track these trends and evaluate the impact of targeted dietary interventions (33).

Firstly, the variability in results could be attributed to the heterogeneity in dietary sources and the composition of omega-6 PUFAs in different studies. For example, arachidonic acid (AA), a type of omega-6 PUFA, has been shown in some studies to promote inflammatory processes that could exacerbate CVD risks. In contrast, linoleic acid (LA), another omega-6 PUFA, is often associated with protective effects against CVD. This discrepancy suggests that not all omega-6 PUFAs uniformly affect CVD risk, and their impact could be context-dependent based on their specific biochemical pathways and interactions within the body.

Secondly, the methodological differences across studies, such as the duration of follow-up, the age and baseline health conditions of participants, and how dietary intake was assessed, can also lead to conflicting results. Longitudinal studies might capture more reliable effects of long-term dietary patterns, whereas cross-sectional studies may not adequately reflect the chronic nature of dietary impacts on cardiovascular health.

Furthermore, genetic factors could play a significant role in modulating the effects of omega-6 PUFAs on CVD risk. Individual genetic variability in fatty acid metabolism might influence how these fats affect the individual’s risk profile. This aspect is often not fully accounted for in many studies, leading to an incomplete picture.

To enhance the credibility and depth of our discussion on this topic, future research should aim to standardize the types of omega-6 PUFAs studied and consider a more detailed analysis of the genetic and metabolic contexts of study populations. Additionally, more rigorous control of dietary assessment methods and a comprehensive evaluation of inflammatory markers could provide clearer insights into the pathways through which omega-6 PUFAs influence CVD risk. Addressing these complexities in a detailed manner would not only resolve the existing controversies but also significantly advance our understanding of the nutritional management of cardiovascular health.

This study has several limitations. First, the time frame of the data used primarily comes from the Global Burden of Disease (GBD) database, which provides epidemiological information for the selected period but does not adequately reflect the latest trends in dietary habits and public health interventions. This may impact the generalizability of the findings. Second, the study lacks data on genetic susceptibility and lifestyle factors (such as physical activity, smoking, and psychological stress), limiting our ability to explore the potential effects of these variables on cardiovascular disease risk. Finally, the analysis does not delve into the molecular mechanisms by which omega-6 polyunsaturated fatty acids (PUFAs) influence cardiovascular disease, restricting our understanding of the mechanisms of action and preventing the formulation of new intervention strategies.

Besides, a notable limitation is the inconsistent data quality and accessibility from less developed areas, particularly Sub-Saharan Africa. Relying on mathematical models to estimate CVD burdens in these regions introduces considerable uncertainty. Moreover, while the study spans from 1990 to 2021, offering a long-term overview, it may not capture the latest dietary shifts or the impact of recent public health initiatives, potentially skewing the study’s findings. This temporal limitation indicates that the latest changes in dietary habits and health policies might not be accurately reflected in the study’s outcomes.

## 5 Conclusion

This study thoroughly analyzes the global influence of cardiovascular disease (CVD) connected to insufficient intake of omega-6 polyunsaturated fatty acids from 1990 to 2021, highlighting a significant decrease over the period. The group most impacted included seniors above the age of 75, with men experiencing a particularly high incidence. Predictions suggest that areas with high socio-demographic index (SDI) values could see decreases in mortality, DALY rates, ASMR, and ASDR in the future. At the national level, places like Russia and regions in North Africa and the Middle East are expected to continue experiencing substantial CVD burdens linked to diets deficient in omega-6 polyunsaturated fatty acids through 2030 and 2050.

These insights are vital for developing CVD prevention tactics and underline the need to regulate dietary patterns.

## Data Availability

The raw data supporting the conclusions of this article will be made available by the authors, without undue reservation.

## Footnotes

^1^ https://www.healthdata.org/research-analysis/diseases-injuries-risks/factsheets/2021-diet-low-omega-6-polyunsaturated-fatty
