# Supplementary material for: Effect of diet low in omega-6 polyunsaturated fatty acids on the global burden of cardiovascular diseases and future trends: evidence from the Global Burden of Disease 2021
Source: Front Med (Lausanne). 2025 Jan 7;11:1485695. doi: 10.3389/fmed.2024.1485695 (PMC11745892; doi:10.3389/fmed.2024.1485695)
Supplement: Supplementary file 1 [file Table_1.DOCX]

Table S1 Global and regional deaths and DALYs of CVD Attributable to Diet Low in Omega-6 Polyunsaturated Fatty Acids in 1990 and 2021 in 204 nations

| Location | Deaths Number in 1990 | Deaths Number in 2021 | ASMR in 2021 | DALY Number in 1990 | DALY Number in 2021 | ASDR in 2021 |
| --- | --- | --- | --- | --- | --- | --- |
| China | 55930.1402 (212352.6398, -170764.5686) | 164339.1363 (649796.9598, -447086.4495) | 9.0647 (35.9068, -24.5349) | 1556556.6382 (5822314.8447, -5115429.4219) | 3388149.9446 (13069419.0685, -9860479.7060) | 172.8301 (669.3736, -505.6742) |
| Democratic People's Republic of Korea | 1073.9425 (4197.0028, -3071.1043) | 2508.8473 (9375.3971, -6373.8644) | 8.3716 (31.6136, -20.9238) | 30513.5728 (121235.7989, -90235.4275) | 64872.1996 (245295.2506, -172526.5096) | 199.2049 (751.0026, -528.0562) |
| Taiwan (Province of China) | 74.2284 (301.0724, -148.8175) | 24.9766 (98.8807, -47.7471) | 0.0586 (0.2316, -0.1150) | 1801.4690 (7084.5208, -3597.1944) | 551.1564 (2085.7262, -1119.2460) | 1.3922 (5.2605, -2.9062) |
| Cambodia | 487.9582 (1836.2831, -1634.4686) | 1137.2593 (4469.6440, -3626.9884) | 10.5153 (40.8398, -32.1577) | 14874.3578 (55064.3932, -53078.8828) | 31383.9991 (122246.6016, -105345.9499) | 241.5306 (949.6051, -785.9104) |
| Indonesia | 9782.2484 (36829.1894, -32381.6214) | 28738.5175 (108769.2369, -91286.6396) | 13.3010 (50.5144, -39.9126) | 315375.7197 (1186264.1237, -1114651.5620) | 851376.9107 (3202382.7201, -2909737.3917) | 323.5447 (1218.1515, -1056.0188) |
| Lao People's Democratic Republic | 469.9417 (1750.2323, -1473.4143) | 715.7072 (2636.2694, -2215.4208) | 17.0335 (63.5907, -50.3778) | 14574.1559 (54196.0289, -48027.6309) | 20811.3289 (76413.9149, -68145.5522) | 403.3476 (1476.9884, -1268.8096) |
| Malaysia | 1418.3480 (5307.6022, -4252.1844) | 3314.0601 (12444.4624, -9718.0022) | 12.4334 (47.1906, -35.7306) | 38328.5450 (140641.1397, -125593.5065) | 88927.2293 (326850.2527, -273168.5863) | 300.3576 (1108.2201, -906.9269) |
| Maldives | 14.1221 (53.3286, -47.1906) | 22.7260 (85.4584, -74.5395) | 7.0807 (26.7651, -21.5792) | 453.0561 (1693.5175, -1589.3351) | 630.1059 (2301.9629, -2211.8265) | 151.6652 (562.0965, -500.0664) |
| Myanmar | 4100.1418 (15859.5369, -13910.7722) | 5665.1241 (21888.3408, -16808.7001) | 12.9434 (49.7821, -37.7962) | 123737.2245 (478320.8077, -443149.6548) | 147459.1077 (570094.2754, -459552.9091) | 294.7468 (1144.9024, -905.5861) |
| Philippines | 4223.3828 (15914.9150, -14729.2017) | 11394.0865 (43054.4838, -38142.6407) | 14.6292 (56.1419, -46.5856) | 132541.7950 (494102.4479, -494603.8253) | 338663.3587 (1280674.9168, -1213537.1984) | 371.5154 (1399.8914, -1285.1048) |
| Sri Lanka | 1286.6386 (4815.8173, -4291.4375) | 2299.7070 (8964.7414, -6629.4755) | 9.0919 (35.1216, -26.2243) | 35985.7869 (132784.5009, -130215.9655) | 57086.1658 (220262.2819, -177261.9380) | 214.6197 (828.5910, -670.6748) |
| Thailand | 2443.5463 (9035.6534, -7940.2017) | 4518.2977 (17432.6647, -13553.6160) | 4.2380 (16.2908, -12.9215) | 67896.3444 (248294.3881, -238659.9479) | 110593.7128 (430746.1273, -355841.8701) | 108.1140 (420.6168, -357.6724) |
| Timor-Leste | 30.2499 (110.4008, -101.1210) | 110.5324 (426.6233, -346.0755) | 14.6027 (55.8134, -44.2025) | 969.3820 (3512.5151, -3536.8273) | 2895.4674 (10965.4121, -9604.1300) | 334.1439 (1273.7956, -1082.6133) |
| Viet Nam | 2314.6890 (8657.1242, -7191.3007) | 6249.1784 (23374.7173, -18126.2404) | 7.0539 (26.2621, -19.6349) | 58321.7360 (213360.8113, -201081.1027) | 152944.9562 (565235.0235, -491655.1857) | 152.1373 (565.0607, -474.0194) |
| Fiji | 37.7720 (140.2236, -87.8817) | 104.5616 (403.5358, -243.8655) | 14.4620 (55.9432, -34.3200) | 1282.8550 (4717.6456, -3074.1189) | 3210.3374 (12014.7424, -7666.3980) | 379.5995 (1441.6990, -895.3742) |
| Kiribati | 7.4063 (27.6047, -25.5089) | 15.4197 (59.7747, -55.2056) | 21.1148 (80.8975, -70.9141) | 247.9403 (923.0856, -904.4706) | 524.6658 (2014.7386, -1960.9353) | 593.5132 (2291.8556, -2144.6637) |
| Marshall Islands | 4.0975 (15.2426, -13.2654) | 9.5577 (36.3994, -33.2306) | 27.6521 (104.0716, -88.9991) | 131.6592 (480.4861, -447.9448) | 328.6361 (1247.7813, -1206.2828) | 753.8662 (2860.5645, -2623.7976) |
| Micronesia (Federated States of) | 12.7762 (48.2097, -42.0384) | 19.2312 (73.5476, -66.9266) | 26.8119 (102.2387, -88.5080) | 401.6366 (1489.0737, -1379.9344) | 624.6308 (2392.9792, -2301.6423) | 729.3843 (2777.6481, -2631.0989) |
| Papua New Guinea | 249.1135 (927.9379, -861.4487) | 742.1532 (2786.9657, -2500.8401) | 14.5706 (55.3263, -44.5012) | 8382.7870 (30944.3764, -30497.3674) | 24845.8465 (93956.5225, -89108.8087) | 388.1833 (1453.5591, -1298.2943) |
| Samoa | 15.3359 (58.7111, -50.8382) | 29.2243 (109.9943, -93.7985) | 21.3843 (81.1897, -65.2562) | 436.9819 (1684.9343, -1541.8277) | 837.5194 (3138.0396, -2916.1197) | 542.8896 (2033.9709, -1846.0574) |
| Solomon Islands | 37.4536 (141.4792, -123.6707) | 92.7464 (342.6819, -327.2032) | 27.6802 (104.7692, -89.5390) | 1201.4663 (4572.9635, -4220.0023) | 3003.5521 (11267.7105, -11414.7817) | 719.3229 (2659.0236, -2548.1878) |
| Tonga | 6.8614 (25.0565, -23.0467) | 10.5001 (40.5637, -30.5604) | 13.2660 (51.5451, -38.3676) | 197.1569 (723.9975, -698.8322) | 278.6529 (1062.1524, -845.0094) | 336.1259 (1280.9503, -1009.5589) |
| Vanuatu | 18.8780 (70.9451, -63.2285) | 51.6463 (193.3258, -175.3699) | 30.5085 (114.1669, -97.9547) | 630.3664 (2369.5559, -2253.9633) | 1695.8391 (6392.2654, -6240.0012) | 818.2580 (3064.5589, -2821.0359) |
| Armenia | 656.8563 (2462.7103, -1970.5712) | 749.4963 (2843.3188, -2079.2156) | 17.5038 (66.2532, -48.8265) | 15371.3963 (56866.0070, -49917.1037) | 15067.9125 (56405.4621, -44279.1879) | 357.2437 (1333.2873, -1063.0406) |
| Azerbaijan | 1492.0106 (5596.3693, -4672.2891) | 2221.8128 (8449.3122, -6487.2615) | 26.3759 (99.5318, -73.4090) | 38184.6307 (140648.7072, -128885.3357) | 52316.8485 (196689.9896, -164448.1851) | 526.8424 (1997.3282, -1581.0593) |
| Georgia | 1860.3027 (7031.2367, -5560.6844) | 681.5668 (2641.4568, -1820.9747) | 11.1412 (42.7915, -30.3876) | 42820.2247 (160269.4472, -138510.4379) | 14672.2037 (55510.2264, -41938.6844) | 262.7692 (984.3046, -771.5997) |
| Kazakhstan | 2941.3614 (10964.7645, -8829.2901) | 2420.4602 (9456.7886, -6585.5812) | 16.9253 (66.5866, -45.2541) | 73186.5346 (268566.3758, -240639.0167) | 52806.0639 (202920.1379, -149371.9095) | 315.0004 (1223.0610, -871.7110) |
| Kyrgyzstan | 641.5689 (2440.0608, -2005.7745) | 934.2819 (3494.8473, -2675.4654) | 23.7021 (90.2943, -65.4555) | 15658.6125 (58409.2322, -53130.4115) | 21590.6636 (78772.3841, -66178.2035) | 462.4902 (1716.4886, -1360.8546) |
| Mongolia | 269.8315 (1026.0624, -798.6769) | 332.1263 (1243.8993, -1006.5127) | 18.4413 (69.9702, -52.6241) | 6521.2167 (24847.5175, -21243.9266) | 8608.6251 (31543.6283, -28553.6549) | 369.0589 (1378.6228, -1130.8902) |
| Tajikistan | 696.1892 (2597.4192, -2114.2130) | 964.9985 (3645.4251, -2692.7435) | 21.1900 (81.1641, -56.6255) | 16941.5799 (62809.6830, -55454.3780) | 24393.0200 (91477.3132, -71592.8819) | 425.5587 (1606.0009, -1193.6987) |
| Turkmenistan | 627.0141 (2351.7604, -1963.6265) | 1098.4443 (4207.3304, -3462.0433) | 31.3176 (120.3570, -94.1023) | 15828.2413 (58681.0814, -52853.0861) | 26946.5339 (102487.6780, -89924.3174) | 666.5102 (2547.0396, -2148.7556) |
| Uzbekistan | 3123.1041 (11750.5480, -9525.3367) | 6478.6708 (24852.2431, -19101.9824) | 29.9353 (114.7732, -84.2016) | 73888.6457 (274035.2780, -244763.3366) | 159831.4790 (600588.3536, -499395.8850) | 614.7094 (2347.1062, -1845.4322) |
| Albania | 271.3212 (1003.2152, -805.0798) | 534.5543 (2067.8503, -1432.9006) | 13.0862 (50.6259, -34.9985) | 6304.6796 (22871.1425, -20295.2647) | 9897.9158 (38178.8209, -28754.7236) | 238.9665 (918.9041, -707.8985) |
| Bosnia and Herzegovina | 659.9763 (2447.0885, -2029.5674) | 730.4585 (2747.8411, -2008.7485) | 11.6597 (43.7279, -32.5127) | 16792.6099 (61204.3782, -55419.7849) | 14029.5676 (52217.6853, -41801.2181) | 232.4319 (864.0053, -713.6383) |
| Bulgaria | 2808.0665 (10746.6811, -7931.6599) | 1747.4091 (6619.0275, -4322.5879) | 12.9921 (48.7879, -32.3777) | 63588.9687 (239699.4272, -192241.8871) | 34515.6106 (129906.7546, -87614.2337) | 276.0497 (1036.7959, -714.1034) |
| Croatia | 1129.1118 (4355.8337, -3086.9431) | 739.4582 (2809.4569, -1764.2220) | 7.7344 (29.3208, -18.7008) | 23569.9905 (88253.0421, -67533.5181) | 11946.2344 (44529.1257, -29532.0320) | 136.7378 (504.9358, -340.7533) |
| Czechia | 3540.7080 (13405.7425, -10307.6903) | 2283.9056 (8686.4554, -6178.8935) | 10.0382 (37.9312, -27.6298) | 78122.6837 (291671.8974, -242059.2803) | 39163.2192 (144873.3051, -112280.2391) | 186.2545 (685.0949, -551.3226) |
| Hungary | 3104.7911 (11727.6511, -9014.1069) | 2279.7544 (8621.1802, -5995.3297) | 11.0295 (41.5470, -29.5681) | 72944.4253 (271486.1784, -229281.6977) | 41644.9810 (154909.5857, -116056.8621) | 222.1163 (816.3039, -632.7237) |
| North Macedonia | 322.0556 (1185.9112, -1012.5931) | 366.1424 (1401.1335, -964.6098) | 14.7080 (55.9184, -38.8497) | 8028.3494 (29393.9099, -26673.9625) | 7825.5918 (29626.4855, -21360.8587) | 265.7238 (1005.1146, -728.2371) |
| Montenegro | 87.8921 (330.2825, -257.7081) | 151.6622 (572.6644, -427.7799) | 17.5856 (66.7935, -49.7174) | 2111.9709 (7880.0846, -6655.6425) | 3028.3041 (11311.8934, -8744.9412) | 330.4605 (1236.9562, -963.8991) |
| Poland | 10223.0883 (38655.6976, -30627.7787) | 7066.3845 (26963.2877, -19386.6384) | 9.3108 (35.5421, -26.0776) | 246461.8159 (911176.4095, -800854.8715) | 129837.1522 (493668.6796, -385952.0448) | 184.8794 (696.2729, -564.5124) |
| Romania | 5355.8462 (20580.7147, -15109.9248) | 4858.5123 (18671.9551, -13728.5058) | 12.4747 (47.7005, -36.0261) | 120039.8304 (450430.4237, -363775.6194) | 92246.4213 (353185.7053, -275034.6539) | 262.4167 (1000.5153, -805.9281) |
| Serbia | 2182.2349 (8234.5778, -6287.3710) | 2297.0536 (9207.1747, -6295.7772) | 13.6151 (54.5439, -37.6123) | 48713.5408 (179570.6903, -150565.1870) | 41550.7165 (164207.1045, -117223.8544) | 259.7117 (1018.6589, -743.0662) |
| Slovakia | 1629.2627 (6190.7760, -4674.6930) | 1254.4559 (4743.1178, -3395.3989) | 13.3338 (50.3509, -36.2794) | 36433.5934 (135164.8789, -111343.1698) | 22844.4177 (85553.2083, -63265.3585) | 246.6649 (918.2792, -691.7070) |
| Slovenia | 259.3890 (983.9563, -729.8435) | 163.5957 (617.5430, -436.0745) | 3.1780 (11.8750, -8.6227) | 5664.1456 (21173.2536, -16957.3351) | 2825.4353 (10473.2595, -7742.4393) | 63.6235 (232.3980, -174.7983) |
| Belarus | 3559.2518 (13505.2251, -10351.5286) | 4513.5378 (16565.6788, -12032.8398) | 27.8754 (102.1102, -74.9462) | 76250.0603 (283260.9483, -236759.5281) | 89752.1408 (326896.0753, -251315.3730) | 574.1872 (2090.0089, -1639.8720) |
| Estonia | 608.4089 (2309.6642, -1716.3759) | 223.6245 (869.6063, -594.7032) | 7.1649 (27.8054, -19.4660) | 12607.8750 (47119.3740, -38269.1750) | 3657.8071 (14113.7633, -10097.6821) | 135.7340 (518.7074, -385.0922) |
| Latvia | 988.5050 (3798.5588, -2804.6263) | 466.4210 (1843.5531, -1142.5927) | 10.6363 (41.5136, -26.3878) | 21343.1223 (80398.4625, -64250.8638) | 8206.3955 (31766.0915, -20543.8975) | 215.9173 (833.1995, -548.1030) |
| Lithuania | 1368.9120 (5220.8902, -3967.0737) | 941.4530 (3674.0492, -2348.9491) | 14.4322 (56.0337, -36.6731) | 28110.2364 (105038.5533, -87304.2654) | 15570.8789 (59710.5637, -40227.3106) | 274.3037 (1037.4906, -729.6601) |
| Republic of Moldova | 1173.9778 (4503.9684, -3408.0661) | 1111.7466 (4211.7497, -3060.8010) | 18.5396 (70.1585, -51.4059) | 25586.1721 (96664.0027, -78560.9623) | 22946.9099 (86259.4060, -67814.5123) | 393.0932 (1471.3933, -1176.0999) |
| Russian Federation | 43866.9521 (166589.4694, -128206.6944) | 43321.4264 (168720.2047, -121827.9916) | 18.2018 (70.6536, -51.6875) | 1030530.7548 (3812691.2974, -3276446.5942) | 913580.7131 (3492710.8329, -2739363.1503) | 396.5114 (1507.3438, -1208.7719) |
| Ukraine | 18642.8139 (70629.0635, -52943.1778) | 24395.1815 (93604.6920, -67220.3191) | 31.0297 (118.8163, -86.5836) | 386619.1325 (1441609.2346, -1183476.3527) | 459891.8596 (1777032.1171, -1335483.7469) | 605.4961 (2328.8406, -1800.4629) |
| Brunei Darussalam | 13.9337 (53.4593, -46.9218) | 25.0952 (92.8828, -81.9179) | 8.0419 (30.2323, -24.0293) | 424.0041 (1601.2825, -1546.5445) | 788.3008 (2834.3831, -2820.2001) | 191.6615 (704.4599, -631.5969) |
| Japan | 7561.3092 (29308.6253, -20244.6105) | 7992.8921 (31199.0839, -19720.8316) | 1.8393 (7.0294, -4.8054) | 154233.8960 (583228.3338, -436051.6727) | 130422.3185 (499738.5409, -338412.7067) | 42.2069 (157.0017, -119.1829) |
| Republic of Korea | 1053.0288 (3966.3484, -3128.8842) | 875.2753 (3456.4310, -1959.7001) | 0.9887 (3.9413, -2.2150) | 29067.0124 (108554.8978, -91955.7759) | 15508.1105 (60269.4186, -34816.7883) | 17.6833 (67.9550, -40.0423) |
| Singapore | 277.2553 (1043.5266, -832.1788) | 294.7380 (1107.0671, -790.5907) | 3.4903 (13.1586, -9.3158) | 7516.4870 (27757.4185, -24226.9473) | 6808.1804 (25005.0945, -19413.5971) | 79.2398 (290.4500, -225.7915) |
| Australia | 2824.0282 (10902.2258, -7946.1037) | 1831.4521 (6999.1091, -5039.4622) | 3.6598 (13.8405, -10.3151) | 57989.0109 (219853.5594, -171784.2776) | 31922.0078 (119792.3546, -93085.5041) | 73.5112 (274.5432, -221.5946) |
| New Zealand | 575.9662 (2217.5938, -1571.5113) | 408.3246 (1564.1670, -1100.5212) | 4.5791 (17.4159, -12.5361) | 12437.7016 (47377.5872, -35648.1276) | 7352.0543 (27453.2542, -20739.1993) | 90.5850 (335.3895, -261.3536) |
| Andorra | 2.6545 (10.2898, -6.7193) | 4.1519 (16.0926, -10.7552) | 2.4149 (9.4358, -6.2105) | 62.5312 (241.6842, -164.0746) | 75.9268 (300.4501, -200.7354) | 48.1526 (191.3294, -128.6065) |
| Austria | 1636.3151 (6282.6986, -4556.3483) | 1118.1623 (4332.6896, -2938.3076) | 5.1913 (19.9803, -13.9318) | 32056.3792 (121667.7418, -94104.1592) | 17893.6604 (68426.6312, -49196.7498) | 96.1836 (362.3993, -275.1288) |
| Belgium | 1217.1015 (4701.2386, -3025.1165) | 475.6524 (1937.3996, -1046.9298) | 1.7635 (7.0215, -3.9409) | 24175.9828 (92197.9419, -61539.1176) | 8242.5581 (32111.3993, -18759.2868) | 36.6476 (139.5807, -85.9744) |
| Cyprus | 47.5470 (188.8134, -103.0854) | 75.5789 (295.8366, -175.9912) | 4.3373 (16.8939, -9.9569) | 943.3452 (3685.4520, -2056.4258) | 1515.7062 (5756.9573, -3645.6374) | 80.9479 (309.0026, -194.4663) |
| Denmark | 1259.5663 (4873.0049, -3272.6322) | 371.6270 (1409.4281, -939.7022) | 2.9038 (10.9258, -7.5392) | 23680.9208 (90037.3988, -63682.3957) | 6631.3010 (24931.4040, -17867.6781) | 59.3919 (220.7466, -167.6424) |
| Finland | 1390.9443 (5282.0882, -3975.4424) | 970.9010 (3760.2547, -2578.8841) | 6.4995 (24.9669, -17.7736) | 29671.0151 (110519.9825, -90890.0616) | 15345.9343 (58377.9002, -42741.4880) | 122.8657 (458.6380, -360.9790) |
| France | 5728.2221 (22157.7977, -15441.6614) | 3553.9151 (13670.5506, -8818.5201) | 1.9998 (7.5586, -5.0877) | 109982.4598 (416131.7216, -319021.0883) | 58010.2992 (217154.5119, -149449.4919) | 41.9575 (153.2321, -111.9816) |
| Germany | 17766.7489 (68955.2275, -46627.6822) | 9137.5588 (35569.0294, -22777.5078) | 4.0811 (15.6993, -10.3561) | 343540.9316 (1305875.9957, -933910.5215) | 150957.5641 (578698.6094, -386171.6986) | 80.0041 (301.1695, -210.4555) |
| Greece | 1757.7581 (6750.5663, -4917.8661) | 1629.6651 (6333.2211, -4354.2997) | 6.0868 (23.3192, -16.9742) | 37180.0689 (139947.1048, -111852.9578) | 29162.4128 (110928.2530, -82867.7250) | 139.1369 (518.3235, -425.1933) |
| Iceland | 37.2831 (142.2144, -98.7735) | 25.7646 (99.3185, -68.0694) | 3.9341 (15.1317, -10.5089) | 729.9724 (2751.3681, -2056.8379) | 441.2586 (1660.7366, -1203.4733) | 77.5447 (287.0909, -214.8315) |
| Ireland | 741.0969 (2852.4335, -2045.0684) | 319.4354 (1233.5369, -843.3243) | 3.8745 (14.8641, -10.3479) | 15621.7497 (59258.8929, -45734.8148) | 5995.0879 (22666.3306, -16688.3566) | 77.9206 (291.7869, -220.8966) |
| Israel | 342.3886 (1353.6720, -785.4374) | 162.9600 (651.3318, -368.9969) | 1.1899 (4.7319, -2.6789) | 6943.2759 (26808.8392, -16142.3217) | 2797.4826 (11054.1769, -6293.2293) | 22.6556 (89.2630, -50.7276) |
| Italy | 6372.7212 (24591.4493, -16245.9091) | 4790.1854 (18220.4371, -11586.4067) | 2.6205 (9.9399, -6.4073) | 129303.6787 (488532.4648, -345911.6855) | 72877.4045 (275585.3168, -178448.9771) | 49.7827 (185.9806, -125.9929) |
| Luxembourg | 63.1037 (241.1443, -169.6741) | 38.9229 (150.3238, -101.7018) | 3.3235 (12.7160, -8.8141) | 1302.1777 (4904.8901, -3667.7008) | 701.2235 (2627.2142, -1938.2044) | 65.3709 (243.3309, -184.6242) |
| Malta | 36.7714 (143.6968, -84.1154) | 40.1253 (154.4439, -97.8507) | 3.8561 (14.7632, -9.4866) | 749.1446 (2882.2854, -1743.7102) | 716.1731 (2720.2549, -1792.4290) | 79.8062 (298.3056, -202.7316) |
| Netherlands | 1522.2835 (5950.6254, -3632.0317) | 721.0387 (2834.0167, -1680.1735) | 1.8969 (7.3689, -4.4466) | 31617.7985 (120323.3376, -77724.5338) | 12576.3226 (47544.8373, -29773.0579) | 37.1679 (139.0719, -89.4873) |
| Norway | 334.2955 (1350.7867, -703.2822) | 125.6917 (495.2159, -271.4322) | 1.1023 (4.3066, -2.3987) | 6095.8150 (24093.4199, -13116.5514) | 2126.9780 (8280.5793, -4630.6864) | 21.3471 (82.7226, -47.1232) |
| Portugal | 1069.4807 (4115.6867, -2844.4093) | 738.7702 (2867.1066, -1877.5292) | 2.7377 (10.4853, -7.1957) | 22590.1996 (85380.1754, -62023.3293) | 13673.1300 (51581.0060, -36895.6691) | 62.0310 (227.9327, -175.9689) |
| Spain | 3932.2906 (15084.1564, -10556.1447) | 2798.9058 (10974.7233, -7032.7036) | 2.4481 (9.4487, -6.3567) | 81015.2468 (302936.1857, -231909.8537) | 49993.5155 (191492.8866, -132929.8769) | 53.8681 (202.3765, -148.4282) |
| Sweden | 2261.2845 (8809.9472, -6006.3179) | 1003.5817 (3900.9526, -2630.0971) | 3.8841 (15.0405, -10.4810) | 40614.2810 (156030.1422, -112683.7382) | 15958.1020 (61556.1194, -44199.8577) | 72.7236 (277.8948, -213.4190) |
| Switzerland | 1297.0116 (5005.4636, -3577.8297) | 707.7479 (2759.4705, -1842.9290) | 3.1091 (12.0197, -8.3145) | 24244.8381 (92004.4213, -71089.9421) | 10711.8837 (40785.7792, -29547.1042) | 56.2392 (211.4868, -162.4320) |
| United Kingdom | 13972.8478 (53870.7625, -37878.4609) | 5220.1986 (20189.9539, -13599.8251) | 3.7563 (14.4001, -9.9744) | 286219.9908 (1083540.6833, -806275.7964) | 97780.2647 (369965.9684, -268557.6590) | 81.3370 (304.2917, -231.2831) |
| Argentina | 3759.6695 (14353.5226, -10456.0522) | 2566.8031 (9736.3659, -6830.0060) | 4.5153 (17.0706, -12.0991) | 86456.1863 (323374.2173, -256667.8345) | 54391.0499 (201800.9904, -152824.3666) | 99.3296 (367.4402, -282.0295) |
| Chile | 825.9308 (3173.3231, -2335.3729) | 752.2662 (2835.6578, -1987.1787) | 2.9187 (10.9716, -7.7595) | 18015.8482 (67745.4824, -53825.3897) | 17408.3821 (63967.9869, -48211.1416) | 69.6472 (255.3345, -194.6153) |
| Uruguay | 492.9920 (1891.2136, -1386.1606) | 209.4670 (804.3848, -500.0430) | 3.4428 (13.0363, -8.2383) | 10584.4149 (39919.2660, -31455.8807) | 3935.1097 (14938.2265, -9455.1575) | 74.9405 (280.0513, -181.0841) |
| Canada | 4241.6221 (16225.9979, -11879.5277) | 3290.1703 (12752.1986, -8873.3181) | 4.2229 (16.2534, -11.6648) | 87833.0523 (327907.6077, -262014.7015) | 59851.6119 (226490.1185, -172170.0433) | 87.2172 (327.2570, -261.7379) |
| United States of America | 15388.1652 (59543.3553, -33449.7720) | 5096.9760 (20489.2730, -9704.1061) | 0.8211 (3.2545, -1.5758) | 310414.7032 (1197469.8494, -675825.5373) | 91328.2076 (364201.5836, -181892.7079) | 16.7375 (66.1200, -33.7178) |
| Antigua and Barbuda | 1.3792 (5.5384, -2.9400) | 2.2418 (8.9035, -4.6955) | 2.3009 (9.2328, -4.7775) | 26.3359 (100.5635, -56.4725) | 50.8194 (195.5968, -111.0974) | 47.8492 (185.7520, -103.5019) |
| Bahamas | 5.1765 (20.1562, -11.2870) | 13.5268 (52.4760, -32.2463) | 3.4570 (13.1909, -8.2401) | 144.9473 (548.7382, -323.5929) | 370.7594 (1421.5177, -920.1579) | 86.1888 (332.2303, -211.3094) |
| Barbados | 21.2548 (81.2753, -53.5960) | 20.2899 (79.5170, -48.8661) | 3.9406 (15.3932, -9.5161) | 421.1524 (1586.6213, -1100.8207) | 413.2156 (1588.6009, -1026.2550) | 82.6526 (316.7544, -207.3242) |
| Belize | 8.5816 (32.3951, -23.1317) | 10.9418 (41.3708, -27.2290) | 3.8954 (14.6848, -9.5440) | 207.9221 (772.5018, -598.9222) | 280.3643 (1030.3308, -743.2160) | 88.4024 (328.2094, -228.5394) |
| Cuba | 1667.5853 (6367.7892, -4702.5793) | 1510.7633 (5717.7985, -3835.6196) | 7.3622 (27.7945, -18.8435) | 36906.6072 (137716.2711, -111163.5837) | 30555.9739 (115010.3105, -80429.9622) | 158.8148 (596.5919, -423.4353) |
| Dominica | 7.0999 (26.9244, -19.3906) | 5.7523 (22.2649, -15.5201) | 7.2789 (28.3089, -19.6330) | 146.9563 (550.4890, -424.5235) | 128.5073 (493.0697, -362.0141) | 154.7635 (594.5186, -434.5491) |
| Dominican Republic | 169.4880 (662.3156, -387.4704) | 155.2061 (627.6658, -312.7494) | 1.5641 (6.4354, -3.1559) | 4621.2493 (16911.3779, -10563.9267) | 3939.4045 (15755.2886, -7972.5105) | 37.9470 (151.8298, -76.4775) |
| Grenada | 7.6239 (28.6801, -19.3981) | 4.4397 (16.9650, -10.3682) | 4.2557 (16.2252, -9.9015) | 176.6623 (655.6239, -471.1304) | 110.6773 (411.1408, -261.1067) | 96.0064 (358.0997, -227.9398) |
| Guyana | 80.3819 (303.4239, -257.4337) | 81.2007 (314.5867, -229.9457) | 13.4119 (52.6511, -37.1117) | 2272.5883 (8551.3669, -7740.9954) | 2210.9018 (8613.3689, -6553.0128) | 325.3499 (1266.9913, -948.8096) |
| Haiti | 617.1014 (2339.0853, -1835.8974) | 965.4790 (3740.3465, -2834.2204) | 15.0755 (59.7493, -42.5154) | 17684.4472 (66727.8558, -54566.0062) | 27735.5571 (107445.2096, -84588.8153) | 351.8272 (1356.8705, -1037.6728) |
| Jamaica | 92.8415 (355.6981, -245.2663) | 117.3620 (462.7695, -301.3535) | 3.5500 (13.9563, -9.1583) | 1945.3674 (7232.9206, -5460.7960) | 2559.2783 (9892.9859, -6846.8876) | 81.1918 (312.8560, -217.4231) |
| Saint Lucia | 9.4892 (36.0935, -27.0090) | 9.3498 (36.1526, -26.2146) | 4.0199 (15.5534, -11.2269) | 212.8490 (796.8941, -658.2108) | 210.2752 (805.3543, -628.6487) | 87.7965 (336.9662, -262.5752) |
| Saint Vincent and the Grenadines | 10.2124 (38.8556, -30.0744) | 10.1994 (38.1811, -28.6198) | 7.9350 (29.5628, -22.0332) | 235.5067 (879.4741, -749.8535) | 226.3969 (856.0156, -669.5987) | 164.6107 (622.3452, -486.1111) |
| Suriname | 24.0468 (92.1201, -59.7945) | 9.4567 (38.8531, -21.2061) | 1.5564 (6.3773, -3.5488) | 642.9743 (2394.5708, -1641.6953) | 236.1926 (952.0819, -502.1474) | 36.7696 (146.8272, -79.4885) |
| Trinidad and Tobago | 46.0673 (184.2484, -97.4497) | 26.9449 (106.1629, -58.2172) | 1.4398 (5.7346, -3.0879) | 1161.0256 (4416.7913, -2511.2264) | 678.5395 (2697.8112, -1464.0528) | 36.2959 (145.0555, -78.3778) |
| Bolivia (Plurinational State of) | 264.4927 (1068.3260, -682.9714) | 424.6274 (1733.3246, -1060.4076) | 5.2569 (21.4601, -12.8231) | 7094.6910 (28264.4024, -18571.7798) | 10495.2429 (42001.9882, -27749.3896) | 113.6948 (459.6464, -293.0738) |
| Ecuador | 319.4610 (1213.0658, -844.6642) | 657.5284 (2591.1806, -1625.7514) | 4.3569 (17.1840, -10.6756) | 8120.4143 (29971.2282, -23021.5143) | 15110.4198 (57674.4019, -39566.0878) | 92.6435 (355.8016, -240.0183) |
| Peru | 763.4966 (2998.5205, -2306.9502) | 1258.0209 (4831.0525, -3634.8119) | 3.7000 (14.2403, -10.6605) | 19499.7429 (74388.3724, -64943.7617) | 30062.2461 (114542.2128, -91497.3673) | 86.5134 (328.2787, -261.1862) |
| Colombia | 2009.3515 (7549.6434, -5946.6528) | 3405.0190 (13253.6386, -8559.5340) | 6.0169 (23.3386, -15.1257) | 52628.1335 (193104.7636, -169471.2266) | 71872.3161 (272588.8100, -185835.9170) | 129.1814 (488.7387, -334.6031) |
| Costa Rica | 165.4559 (629.3814, -472.3137) | 187.5119 (698.3455, -461.5628) | 3.3225 (12.4070, -8.1967) | 3809.4819 (14276.9648, -11663.6984) | 4388.6784 (16010.5881, -11343.8337) | 79.2276 (288.3670, -205.1916) |
| El Salvador | 322.5381 (1221.9059, -1003.9461) | 545.2164 (2111.0287, -1543.9011) | 8.2524 (31.8020, -23.5608) | 8223.1714 (30233.7308, -28466.2068) | 11927.5946 (44817.9166, -36580.5167) | 190.5841 (716.7705, -589.9444) |
| Guatemala | 362.0724 (1375.3421, -1118.4585) | 651.9991 (2498.6095, -1709.9176) | 6.8747 (26.4883, -17.6793) | 10295.9129 (38033.3324, -34789.2721) | 15413.5184 (58187.2458, -42851.9940) | 139.1685 (527.1101, -378.0954) |
| Honduras | 192.4931 (745.9517, -603.5788) | 807.2689 (3055.0374, -2376.7400) | 14.9779 (56.3478, -43.9745) | 5211.3633 (19940.5570, -17694.0091) | 19274.1995 (71547.1192, -58206.5343) | 307.3455 (1147.5546, -909.2119) |
| Mexico | 3023.3900 (11522.7500, -8465.7934) | 10064.6947 (38261.8344, -28394.1462) | 8.5216 (32.5317, -23.6256) | 75236.8832 (276999.6663, -226818.6872) | 237486.1946 (890961.7883, -726795.1318) | 185.6053 (696.9453, -558.7274) |
| Nicaragua | 98.4768 (369.6518, -287.0520) | 267.8743 (1041.3078, -766.4083) | 6.1177 (24.0302, -17.0323) | 2557.4821 (9358.8038, -8242.8627) | 6511.2652 (24285.6015, -19851.7608) | 130.0650 (491.1267, -386.9168) |
| Panama | 46.8385 (183.7379, -104.8071) | 73.0528 (290.4673, -158.8064) | 1.6160 (6.3858, -3.5034) | 1022.4057 (3928.9894, -2295.3100) | 1722.4295 (6612.2046, -3876.0170) | 38.7646 (148.5212, -87.4780) |
| Venezuela (Bolivarian Republic of) | 760.6339 (2861.8485, -1835.8977) | 2063.1725 (8124.2328, -4595.4222) | 7.0652 (27.7560, -15.7700) | 20737.3728 (76414.0156, -52220.2947) | 51505.0905 (198364.5999, -115928.2630) | 168.9563 (651.5427, -380.2884) |
| Brazil | 2472.7428 (9686.6446, -5415.7694) | 4166.2343 (16053.4885, -9471.9602) | 1.6650 (6.4327, -3.7777) | 70379.1012 (267085.3667, -160495.2485) | 113883.7970 (429230.4059, -263822.7370) | 44.5199 (168.2144, -103.0573) |
| Paraguay | 166.3438 (623.8499, -441.9891) | 139.5609 (573.8431, -312.7025) | 2.5028 (10.3291, -5.5760) | 4210.5691 (15447.1589, -12195.8970) | 3292.3837 (13159.9448, -7368.4616) | 55.0091 (222.4777, -123.4449) |
| Algeria | 2210.8582 (8368.2775, -6486.2301) | 4031.2140 (16222.7189, -10540.9319) | 15.0146 (60.8775, -38.1720) | 60051.3674 (224985.5496, -191930.6595) | 94986.6332 (373303.4332, -263877.6982) | 278.1520 (1114.3903, -748.0661) |
| Bahrain | 43.7719 (165.2962, -132.5417) | 69.2007 (258.6020, -189.9964) | 10.7710 (40.6775, -27.5572) | 1344.1977 (4998.6974, -4368.5146) | 2167.0520 (8023.7365, -6185.8600) | 211.5761 (794.6093, -574.1803) |
| Egypt | 7790.4235 (29089.9730, -25529.5371) | 16173.9800 (60634.2742, -49556.9527) | 30.6227 (113.6640, -87.4731) | 232114.0999 (847595.2806, -810026.6090) | 468676.0687 (1756242.8405, -1529300.6333) | 688.7624 (2575.3961, -2119.7596) |
| Iran (Islamic Republic of) | 3439.6245 (12912.2036, -8903.4770) | 5811.5420 (22204.0042, -14749.5457) | 8.1669 (31.6561, -20.1981) | 96359.6004 (357147.5229, -259919.6026) | 141149.5145 (518460.2548, -375026.3150) | 174.3322 (651.7507, -453.9037) |
| Iraq | 1789.1194 (6831.3278, -5338.7968) | 3896.6043 (15131.7159, -10405.9349) | 19.1670 (74.6051, -49.4864) | 48328.1110 (180258.9231, -153496.2625) | 103504.0126 (397055.6899, -283930.0267) | 413.1645 (1598.3039, -1108.8069) |
| Jordan | 203.4509 (769.6121, -627.2082) | 381.4438 (1463.2212, -963.0164) | 5.9809 (22.8441, -14.9330) | 6114.0932 (22943.6681, -19663.3713) | 11056.4131 (42270.6390, -29009.3293) | 132.9373 (508.0700, -340.2894) |
| Kuwait | 72.3898 (265.4327, -186.6330) | 172.5258 (646.6953, -405.8808) | 5.5207 (21.0848, -12.5706) | 2399.7898 (8631.8397, -6455.7219) | 5586.7664 (20633.4890, -13745.8287) | 133.5927 (497.9677, -317.6976) |
| Lebanon | 114.5934 (463.6512, -248.3146) | 234.8958 (930.6576, -543.3006) | 3.6531 (14.5085, -8.5361) | 2899.1790 (11137.3963, -6191.2450) | 4936.9123 (18985.4204, -11967.6079) | 80.4930 (310.2561, -195.5494) |
| Libya | 139.2361 (529.2670, -342.1136) | 403.3715 (1605.7942, -996.5661) | 8.0928 (32.3862, -19.9149) | 3862.1217 (14354.4720, -9900.1080) | 11615.8273 (45575.5959, -29578.8209) | 189.4411 (747.6807, -471.0853) |
| Morocco | 3263.3934 (12516.9456, -9610.6536) | 6003.5000 (23412.2854, -16846.2219) | 19.3100 (73.4912, -53.6387) | 88488.4802 (336529.2770, -281574.3791) | 149276.9492 (594024.7689, -436657.2795) | 426.7655 (1708.1977, -1231.6452) |
| Palestine | 207.7294 (781.9466, -618.4452) | 329.8252 (1235.6689, -1008.3539) | 15.9509 (59.1177, -46.1405) | 5135.1121 (19606.9770, -16741.9867) | 8657.8959 (32594.9231, -28734.5519) | 327.6966 (1228.6091, -1013.7700) |
| Oman | 182.5636 (692.8734, -576.8667) | 265.6227 (1029.3400, -864.2786) | 15.7485 (60.9936, -45.3674) | 5263.5624 (19491.2190, -17593.2791) | 7874.1593 (30038.6218, -27723.8012) | 332.8649 (1287.4618, -1032.4729) |
| Qatar | 21.5574 (82.8108, -61.2269) | 42.2630 (162.2860, -105.2048) | 5.8033 (22.4424, -13.5586) | 710.6462 (2656.1450, -2124.5305) | 1531.6883 (5727.5286, -3897.0143) | 112.7126 (439.4619, -264.5622) |
| Saudi Arabia | 1147.7224 (4213.3785, -3504.7345) | 3819.9830 (14478.8844, -12232.6573) | 17.4347 (66.0444, -50.2698) | 34730.0831 (128505.3599, -112509.5803) | 139238.4841 (521033.4890, -461554.5500) | 455.5980 (1762.6730, -1410.2642) |
| Syrian Arab Republic | 1702.6348 (6407.9916, -5701.1438) | 3281.7096 (12335.8633, -10007.0658) | 29.9145 (113.8249, -87.0371) | 49086.5793 (182070.0843, -175175.2507) | 85614.7166 (323662.0905, -277001.6522) | 643.4259 (2416.1524, -2010.6075) |
| Tunisia | 595.5756 (2280.8308, -1632.4998) | 1380.5925 (5539.2544, -3319.1532) | 11.2848 (45.7702, -27.0459) | 15306.3528 (57517.4494, -44399.8508) | 31910.3230 (126488.5872, -82277.8767) | 241.0138 (953.4087, -612.0874) |
| Turkey | 6168.9518 (23011.5655, -19722.1596) | 9328.2975 (35594.5809, -27696.8806) | 10.7832 (40.8998, -31.4998) | 167900.6812 (626263.1446, -573027.5037) | 206274.0730 (797601.4041, -664665.8525) | 222.0106 (857.3811, -704.3280) |
| United Arab Emirates | 61.6407 (227.1715, -160.6480) | 259.6274 (948.6851, -715.6221) | 10.7418 (40.7984, -26.9563) | 2099.2659 (7668.2141, -5541.1177) | 9261.9449 (33306.9922, -26822.8517) | 204.5305 (774.6213, -527.8159) |
| Yemen | 1458.0206 (5690.0000, -4505.0414) | 3239.5594 (12917.3100, -10941.4298) | 25.1234 (100.4992, -79.7371) | 43917.5443 (171377.5887, -144446.1003) | 93653.5724 (365290.1834, -344773.5030) | 589.8695 (2341.8754, -2002.9391) |
| Afghanistan | 2440.4448 (9400.0450, -7296.9721) | 2666.5206 (10663.2579, -8335.8013) | 27.4954 (107.0450, -81.0015) | 69783.5840 (268129.8786, -216283.8847) | 86068.7270 (340308.6865, -282590.7302) | 689.7100 (2753.1189, -2125.7611) |
| Bangladesh | 5941.7152 (22223.4933, -19409.8373) | 13221.9399 (49788.3198, -39392.3185) | 10.0461 (37.8970, -29.3428) | 187297.5187 (697496.9538, -646936.8292) | 364599.3103 (1379709.6307, -1142888.4437) | 252.4657 (954.0137, -780.4509) |
| Bhutan | 25.2601 (95.8770, -83.3092) | 58.7530 (224.6865, -170.8157) | 10.0626 (38.8201, -28.4904) | 810.4649 (3046.0591, -2863.6789) | 1511.0657 (5772.2357, -4836.7564) | 236.8475 (899.8728, -739.7947) |
| India | 64613.2804 (244853.5189, -227752.7914) | 167367.2478 (622843.2533, -519070.7472) | 14.6641 (55.0095, -43.9323) | 2068545.1310 (7697646.7095, -7694414.2689) | 4741243.5907 (17338346.8569, -15972317.9701) | 374.4218 (1376.9813, -1227.7499) |
| Nepal | 1143.4449 (4195.8963, -3933.8100) | 2343.3061 (8969.3949, -6091.9928) | 10.9804 (42.3438, -28.0072) | 35672.8555 (131831.1525, -128389.2139) | 63699.5732 (244314.4941, -176423.9728) | 263.2568 (1013.0050, -711.0594) |
| Pakistan | 7467.1613 (29437.0504, -24452.5843) | 20927.2814 (81318.5754, -66331.5202) | 17.8469 (70.1736, -53.0686) | 214731.2591 (836834.1700, -768120.5722) | 644175.3479 (2470359.7944, -2193323.1089) | 449.6312 (1742.9066, -1447.2017) |
| Angola | 328.1572 (1285.1232, -1056.1774) | 952.5056 (3651.6827, -2574.6420) | 9.5198 (37.0519, -25.6193) | 9734.2383 (38486.3921, -32040.0360) | 27588.5097 (104727.1406, -76643.2880) | 215.6125 (826.7534, -585.1570) |
| Central African Republic | 168.2152 (661.0328, -568.6343) | 293.7355 (1183.1092, -1002.5521) | 14.7839 (59.5652, -47.5991) | 5033.6880 (19525.0065, -17667.7992) | 9208.6674 (37647.8346, -32815.2725) | 360.1822 (1446.6029, -1216.0875) |
| Congo | 169.0530 (650.6804, -528.9008) | 338.2261 (1310.7604, -1062.1297) | 14.5474 (57.0987, -42.8184) | 4843.7339 (18639.0696, -15855.4885) | 9803.5743 (37633.9502, -32814.7355) | 329.9466 (1276.0480, -1029.4579) |
| Democratic Republic of the Congo | 1639.8042 (6802.1472, -5626.7612) | 3381.7690 (13122.3092, -10447.0831) | 10.7158 (42.1379, -31.5908) | 46129.9094 (191560.9097, -167356.3368) | 96268.5764 (372597.7633, -308107.4689) | 244.5117 (951.0889, -752.8983) |
| Equatorial Guinea | 27.4256 (105.2307, -91.6556) | 56.1948 (225.2117, -172.8335) | 13.0233 (53.4907, -35.6315) | 781.4217 (2986.5750, -2768.1382) | 1565.9127 (6206.9113, -5171.4797) | 283.7631 (1143.0294, -862.1084) |
| Gabon | 67.5284 (265.7673, -201.7944) | 106.1201 (419.9580, -317.4907) | 11.8062 (46.6139, -33.6807) | 1701.1549 (6680.0608, -5363.6459) | 2813.9020 (11047.7068, -9327.3925) | 259.0863 (1024.0885, -795.0858) |
| Burundi | 230.1527 (918.5015, -735.8977) | 373.4619 (1445.7046, -1165.6464) | 8.3401 (32.3197, -24.4250) | 6630.9407 (26140.2410, -22424.7067) | 11350.3852 (42923.4210, -37633.6810) | 201.3004 (773.8078, -626.2748) |
| Comoros | 13.4808 (51.4136, -41.2182) | 31.1958 (119.2526, -88.7542) | 6.9964 (26.5927, -19.1355) | 395.9082 (1483.6345, -1279.8877) | 852.3663 (3227.3515, -2573.4575) | 164.0992 (623.0217, -481.8494) |
| Djibouti | 7.2737 (28.1028, -21.5097) | 45.4570 (180.7192, -137.7079) | 8.0137 (31.6621, -22.7912) | 234.3085 (916.2425, -714.6078) | 1414.3366 (5604.7398, -4422.5364) | 190.0048 (753.2329, -573.7355) |
| Eritrea | 89.2724 (335.6055, -296.2305) | 221.0361 (851.5072, -816.2820) | 8.6657 (32.7937, -27.9509) | 3030.7659 (11297.2680, -10345.4784) | 7154.7929 (27878.4632, -28042.3005) | 213.5408 (815.2167, -767.3305) |
| Ethiopia | 1533.8282 (5716.4583, -5373.9057) | 2101.3389 (7892.9961, -6349.8153) | 5.1972 (19.7134, -14.8083) | 47961.4936 (177470.5390, -178557.3414) | 59732.1031 (221297.5139, -194052.5884) | 122.9827 (458.1686, -379.3200) |
| Kenya | 260.2210 (990.6154, -830.2377) | 1058.7983 (4017.2814, -3458.4118) | 5.3881 (20.7842, -16.3848) | 7221.1943 (27348.7647, -24938.9410) | 30286.3796 (113879.4436, -106513.7647) | 122.8030 (465.9862, -403.8439) |
| Madagascar | 389.4698 (1494.0024, -1252.3215) | 1007.6361 (3841.2671, -3238.2015) | 9.6049 (36.9019, -27.8903) | 11767.4277 (44161.4943, -40025.1751) | 32651.3800 (123576.8385, -114739.8040) | 239.1808 (908.6593, -760.2915) |
| Malawi | 235.7434 (895.3157, -746.9265) | 576.4583 (2174.3156, -1804.7532) | 8.2491 (31.2384, -24.6928) | 7031.8178 (26707.1837, -24083.0948) | 17645.2391 (65247.2900, -58918.1332) | 206.0967 (773.9283, -654.6488) |
| Mauritius | 14.1410 (56.4837, -29.7690) | 17.9377 (69.2719, -34.4262) | 1.0656 (4.1761, -2.0634) | 357.2381 (1380.6781, -733.6597) | 442.8073 (1723.2074, -853.6320) | 26.0651 (100.6566, -50.8036) |
| Mozambique | 161.3999 (603.6361, -474.3590) | 431.2224 (1605.0343, -1267.3329) | 4.3265 (16.0305, -12.3035) | 4620.2754 (17005.0176, -14329.9657) | 13262.5988 (49683.5491, -40836.7855) | 105.6259 (391.0006, -313.9162) |
| Rwanda | 251.3927 (978.6282, -861.2522) | 326.6204 (1289.1249, -1034.6885) | 6.0254 (23.6655, -18.4642) | 7552.3831 (28904.4569, -27338.4907) | 9185.4299 (35701.4114, -30766.0874) | 135.8729 (533.9134, -435.5383) |
| Seychelles | 6.8829 (26.0015, -20.6008) | 8.6471 (32.8876, -26.2001) | 7.9356 (30.6694, -23.3687) | 178.1279 (655.6611, -576.3842) | 230.1635 (844.0178, -767.7166) | 190.6292 (704.0535, -621.0063) |
| Somalia | 145.4958 (568.8082, -464.5530) | 380.5323 (1505.1048, -1252.2636) | 6.6222 (25.8340, -20.7715) | 4832.5482 (18599.9222, -16091.7937) | 12555.2688 (49906.4446, -44177.0773) | 167.5914 (654.8143, -541.3943) |
| United Republic of Tanzania | 638.8684 (2520.7692, -2085.5378) | 2131.1981 (8426.5580, -6532.3710) | 8.9632 (35.2545, -26.0817) | 18178.9715 (71540.3164, -63372.3205) | 60542.4089 (234048.0404, -198283.2487) | 213.5138 (839.8745, -666.0752) |
| Uganda | 389.1739 (1500.0203, -1221.4088) | 876.5364 (3367.5563, -2640.9789) | 6.5253 (24.9105, -18.9458) | 10820.8266 (40507.4913, -36899.6924) | 25633.2178 (99086.0360, -82247.4251) | 154.1204 (588.7699, -468.5735) |
| Zambia | 147.9019 (574.5850, -462.4024) | 530.9215 (2045.5089, -1652.1013) | 8.4935 (32.9994, -24.5489) | 4307.6916 (16234.4422, -14229.4319) | 15804.4780 (60540.9802, -52723.8260) | 199.4905 (770.0616, -617.4775) |
| Botswana | 42.2859 (160.1256, -129.4909) | 83.0132 (311.4844, -249.9828) | 6.4759 (24.6535, -18.6972) | 1202.8804 (4607.5514, -3834.2191) | 2313.4856 (8700.1453, -7377.2231) | 146.8501 (551.1823, -447.0534) |
| Lesotho | 33.6748 (121.0547, -103.1337) | 83.0434 (315.8951, -242.7348) | 8.5775 (32.2177, -24.6356) | 834.9075 (3035.6276, -2683.5589) | 2362.3651 (9432.6864, -7023.5769) | 208.2433 (807.7721, -613.2253) |
| Namibia | 54.6751 (204.8688, -174.0803) | 115.8885 (446.5511, -378.2278) | 9.5421 (36.0454, -29.8847) | 1512.6024 (5636.0676, -5035.6668) | 3180.1707 (12346.8127, -11057.5233) | 218.2363 (842.7658, -725.3404) |
| South Africa | 1284.9521 (4755.6485, -3993.0815) | 2075.0993 (7733.1825, -5665.9193) | 5.0417 (19.0227, -13.4063) | 38730.0049 (141921.1456, -131295.1049) | 55454.9385 (202721.7712, -160373.2818) | 114.9056 (424.1674, -322.9952) |
| Eswatini | 20.8893 (79.0474, -67.1865) | 52.4400 (213.2499, -180.9136) | 10.2125 (41.3901, -31.9179) | 594.3399 (2251.4419, -1974.7251) | 1600.0443 (6402.5025, -5885.8244) | 253.6462 (1022.9358, -880.5134) |
| Zimbabwe | 198.2409 (760.9587, -497.9955) | 489.1041 (1879.8193, -1370.3294) | 8.4431 (32.4494, -22.5665) | 4986.9993 (18673.6025, -13202.8169) | 14143.1774 (54776.0739, -42180.7302) | 191.8612 (738.7802, -540.2099) |
| Benin | 126.7425 (474.3110, -398.4575) | 325.4663 (1219.5837, -1005.3000) | 7.2320 (26.9696, -21.5759) | 3161.2464 (11849.1292, -10640.8533) | 8550.9083 (32358.7506, -28501.4082) | 158.3149 (598.7185, -498.8822) |
| Burkina Faso | 297.9712 (1115.3170, -952.5582) | 725.9961 (2812.9655, -2341.9983) | 9.0798 (35.5756, -27.9166) | 7698.1241 (28522.4261, -26085.4411) | 18188.1923 (70721.8039, -62253.9560) | 194.7198 (746.1171, -644.6067) |
| Cameroon | 288.1038 (1059.0844, -914.0990) | 1052.1830 (4043.5987, -3655.7983) | 9.7426 (37.8351, -31.6059) | 7639.9768 (27733.2880, -25447.5645) | 28864.0276 (110349.5984, -106973.9348) | 215.8250 (827.9382, -753.3375) |
| Cabo Verde | 14.5464 (55.4110, -43.0502) | 34.0324 (130.5315, -86.7745) | 7.9785 (30.9195, -20.2999) | 321.4409 (1214.4416, -1020.0749) | 734.4563 (2815.0283, -1883.9969) | 161.3989 (621.5057, -408.7686) |
| Chad | 214.4013 (846.1709, -709.3443) | 501.2381 (1882.4676, -1719.5099) | 9.8884 (36.9606, -32.0526) | 5320.7295 (20776.3469, -18983.6278) | 13718.3918 (51375.5567, -50536.3937) | 224.5789 (845.9080, -778.0084) |
| C么te d'Ivoire | 378.5393 (1424.3737, -1247.0151) | 1082.0790 (4136.8391, -3695.5844) | 11.2782 (43.2659, -35.1070) | 11055.0440 (41639.8883, -38468.8190) | 30795.6578 (116460.5965, -114917.9354) | 252.2760 (959.2004, -859.3688) |
| Gambia | 30.7846 (114.1192, -98.0082) | 108.3279 (408.8733, -313.9565) | 12.4635 (47.8652, -35.3575) | 835.9358 (3034.5969, -2796.2312) | 2827.7500 (10690.5017, -8606.5150) | 275.9893 (1046.6617, -809.1118) |
| Ghana | 758.0910 (2772.9833, -2398.8878) | 1346.7930 (5077.8722, -4061.1289) | 9.2488 (35.7110, -26.8979) | 21484.4433 (78774.8288, -73063.0733) | 37362.1958 (139005.1715, -118844.5826) | 208.5319 (783.9124, -630.5681) |
| Guinea | 263.2585 (1001.9923, -775.1102) | 520.3990 (1947.4961, -1672.4310) | 10.2708 (38.2527, -31.0848) | 6533.4019 (24929.0568, -20548.9181) | 13475.6636 (50373.5882, -48412.4004) | 229.8822 (858.2261, -769.6509) |
| Guinea-Bissau | 50.3836 (187.1373, -160.6049) | 91.9553 (351.7080, -289.5219) | 14.5035 (56.0173, -43.2983) | 1440.7865 (5401.5053, -4889.8920) | 2771.4037 (10371.3497, -9286.6575) | 339.5535 (1306.4672, -1063.0147) |
| Liberia | 101.6195 (390.0525, -309.0385) | 195.5483 (776.3334, -654.3372) | 10.6035 (41.5045, -32.6661) | 2554.2371 (9673.2532, -8229.8059) | 5498.8210 (21819.0401, -19944.0200) | 235.1039 (931.1465, -788.2309) |
| Mali | 244.5650 (919.6515, -805.3344) | 511.4927 (1957.2557, -1661.4533) | 6.7362 (26.2886, -20.8802) | 6695.9144 (24968.9112, -23533.3636) | 13984.3064 (53071.2795, -47619.5238) | 151.1472 (576.0044, -495.5999) |
| Mauritania | 100.3218 (375.6087, -254.2753) | 158.2148 (630.3928, -428.7656) | 8.3695 (33.4021, -22.3668) | 2538.6098 (9309.4929, -6808.0896) | 3821.8674 (15117.2328, -11018.6579) | 175.9436 (701.0715, -487.1873) |
| Niger | 148.7489 (583.2723, -494.4787) | 458.7040 (1741.2158, -1563.1504) | 6.7448 (25.6608, -21.7041) | 4100.9594 (15836.2316, -14105.1644) | 12149.0871 (45879.4862, -43776.9209) | 146.0895 (553.7269, -500.4996) |
| Nigeria | 3853.1204 (14699.6597, -11645.0938) | 7560.5124 (27656.9415, -24749.4148) | 9.9254 (36.3801, -30.2337) | 96887.6141 (366488.1520, -308378.5141) | 194761.5184 (714501.0392, -692554.2507) | 206.4574 (754.4041, -685.0677) |
| Sao Tome and Principe | 4.1518 (15.5595, -11.8868) | 8.9137 (33.3989, -26.0118) | 9.1748 (35.1066, -25.9484) | 98.9347 (367.2651, -301.3484) | 235.6968 (867.3752, -714.1203) | 200.0185 (745.8013, -586.5041) |
| Senegal | 310.8784 (1175.1458, -953.7069) | 599.4701 (2348.7107, -1653.3071) | 8.9246 (34.9972, -23.4890) | 8096.1415 (29831.1597, -25788.3450) | 15042.3745 (58040.3082, -44768.7321) | 190.7039 (746.1622, -543.0035) |
| Sierra Leone | 227.0668 (867.2525, -712.0884) | 414.0892 (1581.2451, -1247.1978) | 12.2624 (46.5684, -35.2266) | 5641.7598 (20879.3445, -18834.8195) | 11070.6412 (42727.2524, -35721.0666) | 275.7315 (1059.0262, -846.2619) |
| Togo | 115.1663 (427.2090, -361.2295) | 359.0570 (1384.1455, -1158.5670) | 11.0252 (42.9687, -32.4797) | 3166.0381 (11652.3856, -10472.5122) | 10180.8002 (39011.7581, -35784.8642) | 247.7846 (953.0314, -801.0351) |
| American Samoa | 3.1628 (11.6023, -9.8132) | 7.5567 (28.0104, -23.1687) | 16.6448 (61.8249, -49.6064) | 102.6195 (376.1139, -333.9031) | 221.7685 (813.6987, -714.0880) | 435.6245 (1598.3292, -1374.5106) |
| Bermuda | 8.6951 (33.6254, -22.3200) | 6.2010 (24.5612, -15.2603) | 4.2791 (16.8457, -10.6476) | 197.7582 (751.1200, -522.8840) | 120.7938 (459.1618, -303.7068) | 93.4703 (352.2009, -240.0112) |
| Cook Islands | 1.7295 (6.4578, -5.1422) | 2.2860 (8.6213, -6.6158) | 9.2683 (34.9456, -26.9283) | 50.4577 (186.3097, -158.8123) | 56.3544 (211.5940, -173.2495) | 234.6705 (885.0449, -726.0018) |
| Greenland | 4.0436 (15.0917, -10.6016) | 2.3374 (9.1284, -5.5475) | 3.7592 (14.8930, -8.9294) | 120.8235 (449.2725, -325.3339) | 62.3086 (240.7497, -150.3012) | 87.1961 (334.9978, -216.0897) |
| Guam | 9.8882 (36.3023, -28.6254) | 24.2115 (88.9194, -68.4597) | 11.5720 (42.4829, -33.0177) | 293.7465 (1065.9538, -887.0764) | 700.2066 (2545.9924, -2082.4604) | 351.0913 (1273.3371, -1064.8357) |
| Monaco | 2.5710 (10.0565, -5.5262) | 1.2143 (4.8257, -2.5646) | 1.0925 (4.2497, -2.3001) | 46.7053 (180.7978, -105.5270) | 20.2584 (76.3916, -42.9131) | 22.9003 (85.9111, -49.1653) |
| Nauru | 1.8937 (7.1099, -5.9618) | 2.5628 (9.9877, -8.8121) | 43.1036 (167.8421, -139.1719) | 66.6897 (247.8814, -217.4550) | 88.3450 (343.6567, -318.4995) | 1220.0280 (4751.6662, -4226.5051) |
| Niue | 0.5138 (1.9554, -1.5428) | 0.4653 (1.7396, -1.2805) | 22.3725 (83.7783, -61.6764) | 12.3818 (46.5595, -39.9417) | 11.7464 (43.8309, -34.2057) | 557.0832 (2082.2425, -1643.1508) |
| Northern Mariana Islands | 2.0840 (7.5374, -6.1308) | 6.3026 (23.6058, -19.0246) | 13.1038 (49.7641, -38.4264) | 77.9061 (280.3010, -234.8240) | 190.5471 (702.3868, -595.9730) | 332.2002 (1227.0486, -1028.8499) |
| Palau | 2.1802 (8.2003, -6.7784) | 4.4006 (16.5796, -13.6462) | 21.7472 (81.7199, -65.1433) | 67.3414 (252.1902, -223.8862) | 136.3830 (504.3283, -441.9242) | 570.9152 (2123.7443, -1829.2677) |
| Puerto Rico | 294.6056 (1155.0525, -726.6766) | 166.5545 (636.3891, -355.7922) | 2.1454 (8.0406, -4.7045) | 6496.2451 (24490.3443, -16497.8097) | 3272.8210 (12298.1282, -7176.7653) | 52.4277 (195.5253, -121.5943) |
| Saint Kitts and Nevis | 5.2557 (20.2038, -13.2946) | 2.9183 (11.4255, -7.2051) | 4.9112 (19.2568, -11.9185) | 115.6587 (432.0761, -302.3527) | 72.7021 (284.0291, -184.1126) | 104.0539 (409.3747, -260.6954) |
| San Marino | 1.8049 (7.1407, -4.5936) | 1.4272 (5.7442, -3.6330) | 1.4864 (5.9410, -3.8778) | 32.8955 (128.1597, -87.7568) | 23.5159 (92.3784, -63.7631) | 30.9778 (123.8848, -88.8207) |
| Tokelau | 0.2756 (1.0409, -0.8235) | 0.2843 (1.0949, -0.7858) | 19.4725 (74.9737, -54.1865) | 7.1240 (27.2605, -22.3545) | 7.0584 (26.5083, -20.9936) | 489.6467 (1841.3750, -1471.7805) |
| Tuvalu | 1.7008 (6.3010, -5.7896) | 2.6004 (9.5741, -8.1016) | 26.7370 (100.5442, -81.1629) | 52.2539 (193.7114, -187.0240) | 76.8721 (287.1464, -252.9897) | 711.5092 (2659.7450, -2310.9176) |
| United States Virgin Islands | 8.1560 (30.8185, -20.8518) | 5.1819 (20.7687, -10.8382) | 3.1597 (12.5506, -6.5533) | 215.0468 (789.1956, -555.1709) | 107.9167 (439.3283, -233.9277) | 69.8050 (283.2616, -154.8896) |
| South Sudan | 166.2679 (662.7130, -507.0078) | 278.4187 (1113.7061, -946.8838) | 7.7579 (31.2411, -24.0122) | 4626.0762 (18345.1816, -14878.8993) | 8456.6875 (33275.2447, -30582.7840) | 190.4510 (761.8852, -642.7508) |
| Sudan | 2821.8421 (10722.8596, -8886.7726) | 4267.5208 (16983.2458, -12076.0674) | 23.1819 (91.5956, -61.9836) | 82203.1706 (309893.4790, -285230.4665) | 123503.8885 (485705.2811, -383575.3920) | 549.8632 (2188.7703, -1577.7762) |
